# Advances in Titanium-Based Biomaterial for Human Bone Scaffolds: Narrative Review on Design, Fabrication, Surface Engineering, Implantation, and Biological Evaluation

**DOI:** 10.3390/ma18235421

**Published:** 2025-12-01

**Authors:** Sichale W. Fita, Mirosław Bonek, Anna Woźniak, Sebastian Sławski

**Affiliations:** 1 Department of Engineering Materials and Biomaterials, Faculty of Mechanical Engineering, Silesian University of Technology, 44-100 Gliwice, Poland; 2 Department of Materials Research Laboratory, Faculty of Mechanical Engineering, Silesian University of Technology, 44-100 Gliwice, Poland; 3 Department of Theoretical and Applied Mechanics, Faculty of Mechanical Engineering, Silesian University of Technology, 44-100 Gliwice, Poland

**Keywords:** biomaterials, titanium, scaffold, additive manufacturing, surface modification, osteointegration

## Abstract

The growing demand for reliable orthopedic implants has driven extensive research into biomaterials and metal alloys for the development of bone scaffolds. This review summarizes current progress in improving scaffold performance by optimizing mechanical strength, biocompatibility, and bone integration. Key studies on material choice, modeling methods, manufacturing techniques, and surface treatments are discussed, with a special focus on titanium-based alloys due to their favorable mechanical and biological properties. Computational tools, particularly finite element modeling, are increasingly used alongside experimental findings to illustrate mechanical behavior and to guide design of structures that more closely resemble natural bone. Both additive and traditional manufacturing routes are considered, emphasizing how porosity, geometry, and fabrication parameters affect mechanical stability and tissue response. Surface modification approaches, both physical and chemical can enhance cell attachment and antimicrobial function. Overall, this paper shows how combining materials science, mechanical analysis, and biological testing helps develop bone scaffolds that offer durable mechanical support and clinical outcomes.

## 1. Introduction

### 1.1. Introduction to Biomaterials and Metal Alloys for Bone Scaffolds

Biomaterials are materials, either natural, (derived from living organisms, such as plants or animals, like protein, gelatin, alginate, silk, fibrin, cellulose, chitin, chitosan) or synthetic (synthesized in laboratory-like polymer, metal, and ceramic), used in medical devices, collaborating with biological systems for therapeutic or diagnostic purposes. Additionally, they are designed for use with biological systems in medical applications, including the treatment, improvement, repair, or replacement of tissue functions within the body [1]. The first use of biomaterials started with Neanderthals’ use of wood as a dental implant material [2]. From 7 BC to 4 AD, ancient civilizations, including Greece and Rome, utilized several naturally occurring substances and metals to treat wounds and other medical problems [3,4]. During the 16th century in Europe, dental repairers used silver and gold materials, whereas iron threads were used for bone repairs and various immobilization procedures [5].

In the late 19th century, technological advances in X-rays, anesthesia, and sterile surgery led to the use of metals for internal body repairs [6,7]. However, the use of those metals leads to more difficulties than solutions. The main causes of these issues were corrosion and inadequate mechanical properties that prevented the products from performing their intended purpose [8]. Especially during the 1960s, problems related to the presence of certain implants started to be determined. As a result of this issue, the field of biomaterials recognized the need for engineers to develop implants and substitutes for damaged body parts in the late 1960s. Engineers began working in medical, surgical, and dental clinics, contributing to biomedical literature [9,10]. The first Biomaterials Symposium at Clemson University in 1969 marked the incorporation of auxiliary fields of engineering and medicine for biomedical material development [11].

**Figure 1:**
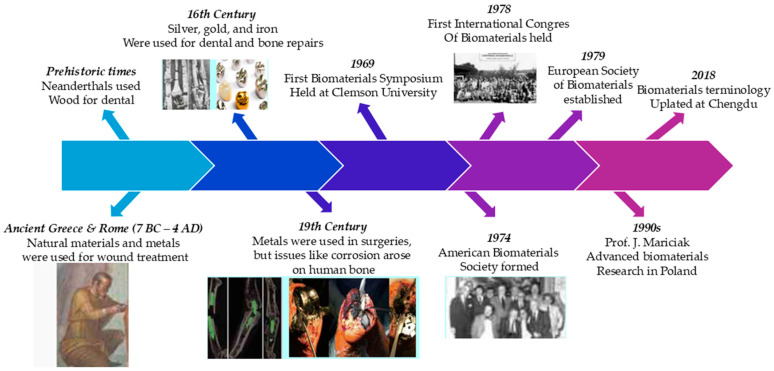
Timeline summarizing key historical milestones in the development and application of biomaterials. The author created the figure based on ideas synthesized from multiple literature sources [1,2,3,4,5,6,7,8,9,10,11,12,13]. No copyrighted images were reproduced.

Meanwhile, the study of biomaterials has been divided into different societies, such as the American Society for Biomaterials (established in 1974) and the European Society of Biomaterials (established in 1979). The first International Congress of Biomaterials in 1978 suggested the start of a worldwide field committed to replacing damaged human body parts [12]. The 1986 lexicon, initially published in 1987, was used until 2018. At the 2018 Chengdu Conference, biomaterials terminology was revised to reflect advancements in the field and add new terms. These new definitions were issued in 2019 [13]. To conclude, the history of biomaterials is generally presented in Figure 1.

**Figure 2:**
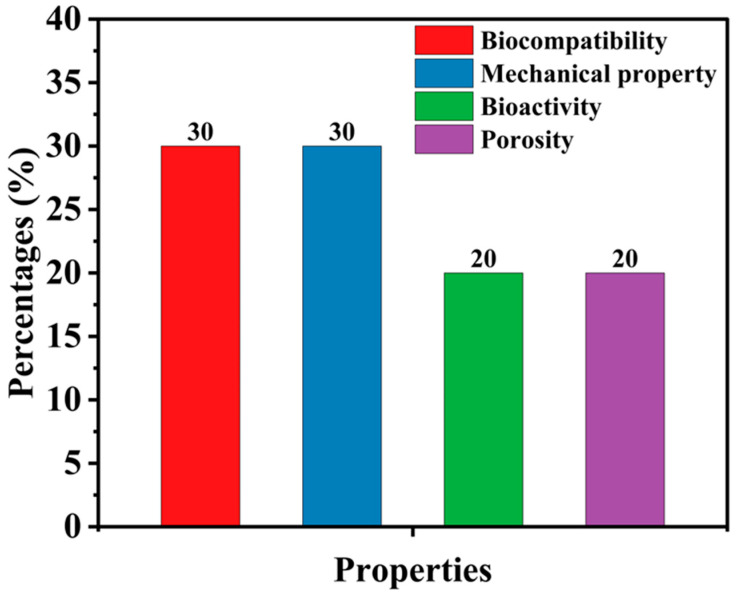
Composition prioritization in bone scaffold preparation [25].

**Figure 3:**
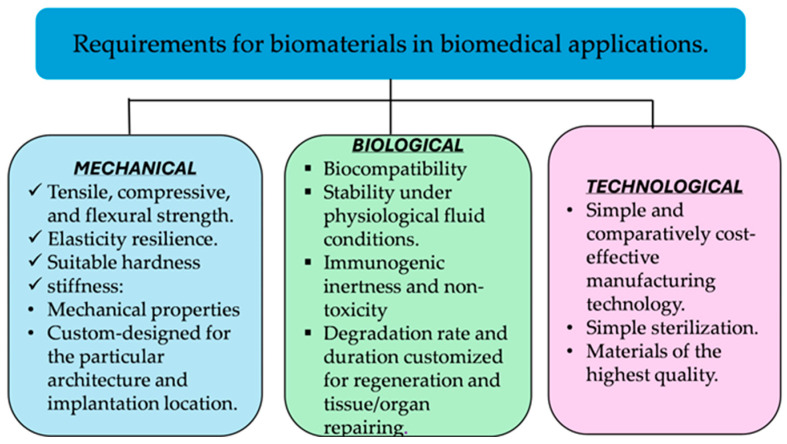
Key requirements for biomaterials in biomedical applications (adapted from Kurowiak et al. [26]).

Biomaterials to be used for human bone replacement should have mechanical properties such as high toughness [14], specific strength, and a Young’s modulus that closely approximates that of natural bone [15,16]. In addition, they should exhibit biocompatibility [17,18], bioactivity [19], and, in the case of temporary implants, controlled biodegradability [19,20]. For practical use, a balanced percentage of these parameters is recommended for the preparation of bone scaffolds [21]. Selecting appropriate biomaterials for human bone applications requires a comparative assessment of critical design parameters. According to published studies’ review of the literature [17,22,23,24,25], biocompatibility and mechanical properties are identified as the most crucial parameters in biomaterial design, with each attribute accounting for approximately 30% of the overall consideration. This underscores the consensus among researchers that an ideal biomaterial must ensure biological safety while also exhibiting sufficient mechanical integrity to withstand physiological loads. In comparison, bioactivity and porosity are attributed a relative significance of 20% each. Bioactivity, defined as the material’s capability to interact positively with biological tissues, plays a vital role in promoting cellular attachment, proliferation, and integration with host tissue. Porosity, particularly relevant in the context of scaffold fabrication, is essential for enabling nutrient diffusion, waste removal, and tissue ingrowth. Figure 2 presents the relative percentage contributions of key factors influencing bone scaffold design. To determine the suitability of various materials for human bone applications, Table 1 presents a comparative analysis of the properties of commonly used biomaterials. Additionally, biomaterials used in biomedical applications have to meet specific mechanical, biological, and technological requirements to ensure their functionality, durability, and compatibility with the human body, as illustrated in Figure 3.

**Table 1:** Biomaterial properties (metals, biodegradable polymers, natural polymers, and composites) for bone scaffold preparation.

Class	Material	Young’s Modulus (GPa)	Tensile Strength (MPa)	Biocompatibility	Source
Metallic	Ti-40Nb Alloy	30–50	_	Excellent	[27]
	TiAlV	110–120	_	High	[28]
	Ti-10Mn Alloy	76	860	Low	[29]
	Co-Cr-Mo Alloy	200–250	900–1540	Low	[30]
	316L Stainless Steel	190–210	540–1000	Low	[31]
	Mg-Based Alloys	150–400	150–400	High	[32]
Ceramic	β-Tricalcium Phosphate (β-TCP)	5–10	_	High	[33]
	Bioactive Glass (45S5)	35–50	_	High	[34]
	Zirconia (ZrO_2_)	200–210	_	High	[35]
	Keratin/Hydroxyapatite	_	_	High	[36]
	HA/HDPE Composite			High	[37]
	Mg-Ca/β-TCP composite	45	>300	High	[38]
Polymer (Synthetic)	PLA/PLGA (Poly-lactic/glycolic acid)	1–3	50–70	High	[39]
	PCL composites	0.004–3 Adjustable	Variable	High	[40]
	HA/PCL/gelatin composite	0.0045	1.93	High	[41]
Polymer (Natural)	Silk Fibroin Composites	5–15	_	High	[42]
	Chitosan composites	5.2–100	_	High	[43]

Biomaterials are specially engineered to interact with biological systems, playing a crucial role in medical applications. They encompass a wide range of materials, including metals, ceramics, polymers, and composites, each chosen for its unique properties tailored to specific biomedical uses. From those biomaterials, Ceramics (for example, hydroxyaptite and bio-glass) are used due to their bioactivity and compatibility with bone tissue, making them beneficial for applications in bone grafts and as coatings on metallic implants [44]. Additionally, polymers such as polylactic acid (PLA) and polyethylene (PE) are frequently used in various biomedical applications. PLA is particularly suitable for use in drug delivery, sutures, and tissue engineering scaffolds because of its biodegradability and mechanical strength, whereas polyethylene is preferred for long-term implants due to its excellent durability and stability in the body [45]. While composites, which integrate the properties of various materials, enhance functionality and find applications in advanced fields such as tissue regeneration scaffolds [46].

Biomaterials, like metals, ceramics, polymers, and composites, play an essential role in medicine by offering specialized properties suited to interact with biological systems. Specifically, metals such as titanium and stainless steel are frequently applied in orthopedic implants because of their excellent mechanical strength and resistance to corrosion, making them suitable for load-bearing applications [47]. Their unique mechanical, chemical, and biological features make them ideal for a wide range of applications.

However, the application of biomaterials as scaffolds in bone tissue engineering presents challenges, mainly concerning their mechanical properties, biocompatibility, and the complex processes of bone regeneration. According to Ansari et al. [48], human bone scaffolds must be strong enough to support physiological loads and porous sufficient for cell infiltration and vascularization. Moreover, to support cell attachment and differentiation, biomaterials must exhibit biocompatibility and promote integration by mimicking the natural bone environment [49]. Another key challenge in biomaterials development is designing scaffolds that both initiate bone regeneration [19] and have their property to degrade appropriately over time. These challenges require innovative solutions to enhance scaffold effectiveness and its compatibility with surrounding tissues.

### 1.2. Titanium Alloy Use for Human Bone

Titanium was separated in the early 19th century, but its alloys became popular in the mid-20th century for their excellent strength-to-weight ratios and corrosion resistance in aerospace applications [50]. Even though it was energy-intensive and expensive, the 1940s Kroll process became standard for titanium extraction, and this was the first time that the application of commercially pure titanium (CP-Ti) in medicine started [51]. Since the early 1970s, titanium and its alloys have been widely used in biomedical applications, including the production of implants and the treatment of bone defects. These alloys have a reduced Young’s modulus in comparison with other materials, like stainless steel, cobalt-chromium alloys, and tantalum [52], which makes them more appropriate for biomaterial use. Furthermore, titanium’s biocompatibility, non-toxicity, and capacity for osseointegration make it one of the most suitable materials for long-term implantation into the human body.

Titanium (Ti), a transition metal with the atomic number 22, is recognized for its remarkable characteristics and adaptability across a range of applications. It has a density of about 4.54 g/cm^3^ and a melting point of 1668 °C. The yield strength of some alloys ranges from 470 to 1060 MPa, while this metal is known for its excellent biocompatibility [53,54], corrosion resistance [55], high strength-to-weight ratio [56], and mechanical strength [57] which makes it useful for various medical applications and human bone implantation. However, challenges like the possibility of bone resorption and implant loosening due to stress shielding are the main problems that current researchers need to resolve, thereby confirming titanium as a top option for biological applications.

Titanium exhibits two allotropic phases: hexagonal-close-packed crystal structure (HCP) alpha (α) and body-centred-cubic (BCC) beta (β). The transformation from the α-phase to the β-phase occurs at around 882 °C [58]. These phases differ in their crystal structures and respond differently to alloying elements, which significantly influence the mechanical properties and potential applications of titanium in various fields. Titanium alloys are classified into three main types, depending on their microstructure: single-phase alloys (α, or β) and dual-phase (α + β) alloys, each possessing unique properties and used in different areas.

**Figure 4:**
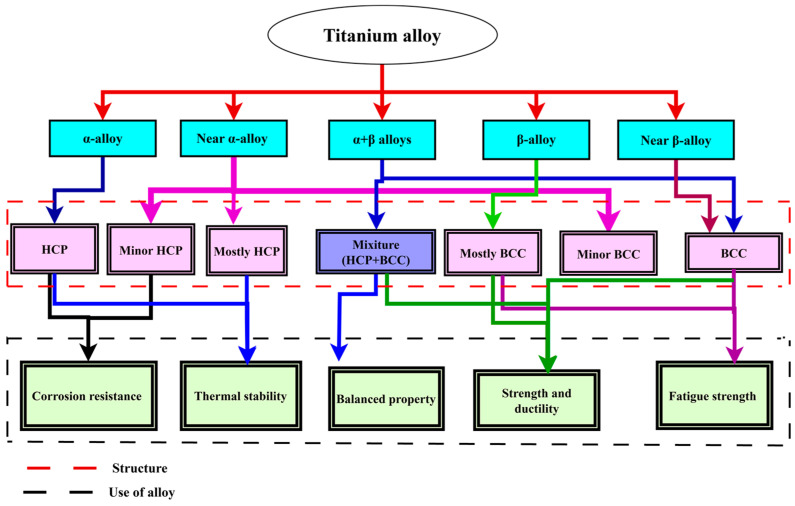
Chemical composition, alloy type, structure, and advantages of titanium alloy for human bone.

Alpha (α) alloy is known for its excellent corrosion resistance and good weldability, while beta (β) is known for its excellent strength and toughness, which is improved after heat treatment. In the same manner, α + β alloys offer a well-balanced mix of mechanical strength, flexibility, and resistance to corrosion, which makes them ideal for use in both structural and biomedical fields. On the other hand, alpha (α) alloy is suitable for high-temperature applications due to its stability at high temperatures. While beta (β) alloy is widely used in applications requiring high strength and flexibility, and alpha-beta (α + β) is commonly used in aerospace for structural components due to its excellent characteristics [59]. Generally, the structure and advantages of Titanium alloy for human bone are illustrated in Figure 4.

**Figure 5:**
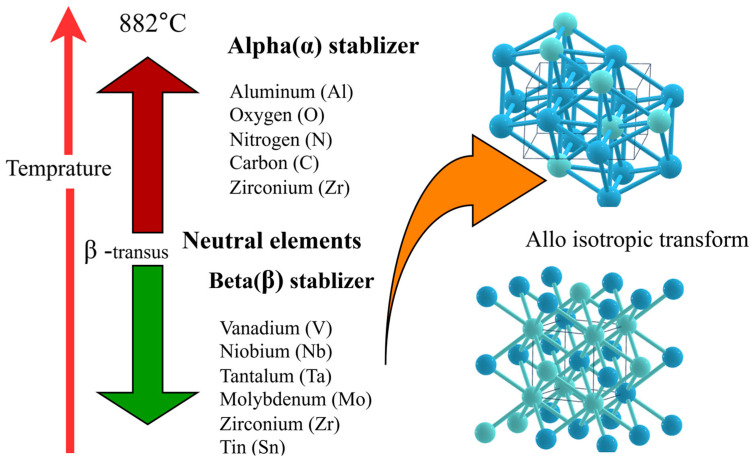
Categorization of titanium alloy stabilizing elements based on their ability to stabilize either the α or β phase.

The stabilization of α and β phases in titanium alloys is monitored by several alloying elements, each with unique effects on phase stability and mechanical properties. Aluminium, vanadium, molybdenum, niobium, tantalum, and tungsten are the main stabilizers. In alloys like Ti-6Al-4V, aluminium (Al) stabilises the α phase, increasing strength and ductility. However, excessive use of Al causes a coarse-grained structure and reduces ductility [60]. Likewise, vanadium (V) stabilises β and causes β to convert into α’ martensite at specified concentrations and high cooling rates during the fabrication process (for example, a 3D printing method). Increasing the stability of the β phase results in brittleness if overused [61]. While molybdenum (Mo) and tungsten (W) stabilize the β phase, preventing secondary phases like α’ and ω from precipitation [62]. Similarly, niobium (Nb) and tantalum (Ta) stabilize the β phase but may cause metastability. Nb may coexist with ω and α phases, while Ta increases ω precipitation [63]. Generally, the addition of β stabilizers increases strength and ductility in fine-grained structures, while excessive amounts in coarse-grained structures can cause irregular plastic deformation [64]. To visualize these stabilizers, Figure 5 illustrates the titanium alloy stabilizing elements and the associated structural transformation. Generally, although stabilizers improve phase stability and mechanical properties, overuse can lead to brittleness or decreased ductility.

Pure titanium (CP-Ti) is classified into grades 1 to 4 (including 4a and 4b), exhibiting enhanced strength and decreased ductility. It promotes osseointegration and is biocompatible, making it suitable for orthopedic implants [53]. While the most popular titanium alloys, such as an aluminium–vanadium alloy, like Ti-6Al-4V (containing 90% titanium, 6% aluminum, and 4% vanadium), are known for their high strength-to-weight ratio and remarkable fatigue resistance, making them suitable for load-bearing implants and often used in orthopedic implants [50]. Even though aluminium and vanadium toxicity led to the development of alternatives, of aluminium-vanadium-free elements like Ti-6Al-7Nb, the alloy, which decreases toxicity while maintaining mechanical properties [65].

Generally, titanium alloys are essential in medical treatments due to their exceptional qualities, making them suitable for a wide range of biomedical applications. Their distinctive combination of biocompatibility, mechanical strength, and corrosion resistance makes them safer and more efficient for medical treatments. For instance, they are widely used in hip joints [66], fixation screws for femoral prostheses [56], spine, long bones [67], femur, tibia, and knee implantation [68] and other parts of the human body. On the other hand, titanium alloys are used in vascular stents due to their biocompatibility and mechanical properties [69]. While specialized alloys, like superelastic and shape-memory alloys, have been created for specific applications such as reconstructive surgery [53].

**Figure 6:**
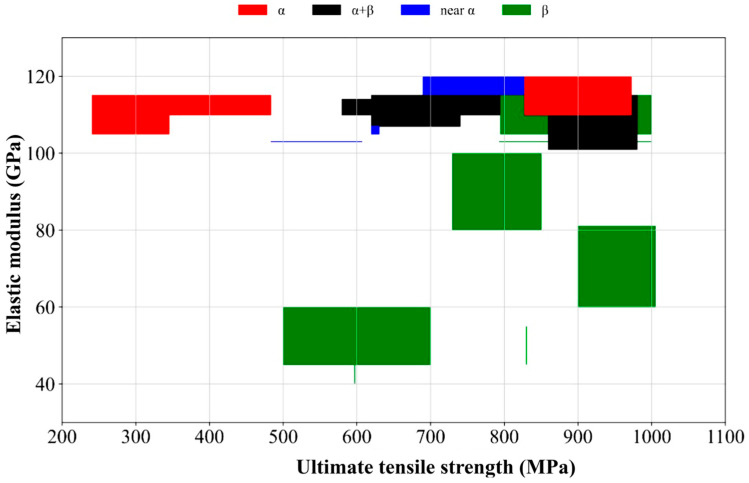
The relationship between mechanical properties and microstructure of different titanium alloys.

Based on previously published research, Table 2 outlines the mechanical properties of biomedical titanium, its grades, and the various structure types. Figure 6 illustrates the relationship between structural behaviour and Young’s modulus and tensile strength, which shows the mechanical properties of various titanium alloy types, including α, near-α, α + β, and β, based on their ultimate tensile strength and elastic modulus. α alloys exhibit high stiffness but relatively low strength, whereas β alloys show greater strength with lower stiffness, making them ideal for applications that need both strength and flexibility. The near-α and α + β alloys strike a balance between strength and stiffness, offering versatility for a wide range of engineering and biomedical uses. Furthermore, Figure 6 indicates two distinct α-type clusters. The commercially pure α-titanium grades 1–4 with coarse grains and low interstitial content fall into the lower-strength range (240–550 MPa), while strengthened α or near-α alloys such as Ti-5Al-2.5Sn (Grade 16) drop into the higher-strength range (828–972 MPa), where alloying and refined α microstructure provide much higher tensile strength.

The alloying compositions and mechanical properties of the titanium alloys are listed in Table 2, and Figure 6 shows how these compositions influence the structural type and ensuing mechanical behavior. Each alloy group’s position in Figure 6 reflects the function of the alloying elements listed in Table 2. Al, Sn, Ni, Pd, or Ru are commonly found in α-type alloys, which possess a high modulus (103–115 GPa) with a lower strength. The addition of β-stabilizing elements, such as V, Mo, Cr, or Nb, leads the alloys to transition toward near-α and α + β structures, increasing their strength (up to about 900 MPa) and slightly decreasing their stiffness. Fully β-type alloys rich in Mo, Nb, Ta, or Zr generate single-phase β microstructures with a reduced modulus (40–90 GPa) and a high tensile strength (up to 1000 MPa). These composition-dependent trends demonstrate how titanium alloys become systematically stronger and less stiff as the β-stabilizer component is increased. This combination is suitable for biomedical implants that require durability and mechanical compatibility with bone.

Titanium exhibited good corrosion resistance (the best in the metal biomaterials group for long-term applications) due to its ability to spontaneously form a thin oxide layer in air or water. Creation of the passive layer is influenced by the alloy composition, with specific components like Mo, Ta, and Zr in β-Ti alloys increasing the layer’s protection qualities [82]. In general, the passive oxide layer significantly reduces the corrosion rate by acting as a physical barrier to the electron transfer processes that are necessary for metal failure. Studies in simulated body fluids have shown that alloys like Ti-6Al-7Nb exhibit excellent corrosion resistance, primarily due to the formation of a stable passive oxide layer, which makes them well-suited for biomedical implant applications [83]. Additionally, the long-term stability of the oxide layer is crucial in environments with varying pH levels, such as the human body, as it prevents crevice corrosion and hydrogen embrittlement. However, the presence of alloying elements can cause toxicity if they leach into the body, necessitating careful selection and design of titanium alloys for biomedical applications [84]. Generally, a passive oxide layer offers considerable corrosion protection, although its effectiveness can vary depending on the environment and alloy composition.

The biocompatibility of metallic biomaterials can be enhanced through three primary strategies: adjusting the chemical composition, applying heat treatment, and utilizing surface modification techniques. Surface modifications are typically classified as mechanical (e.g., grinding, laser texturing), chemical, or physical. These techniques aim to improve interactions between the material and biological tissues. For example, laser-textured titanium surfaces exhibit increased roughness and super-hydrophilicity, which promotes protein adsorption and cell attachment; however, excessive hydrophilicity may impair cell function [85]. Incorporating elements like calcium and phosphorus via laser treatment has also been shown to enhance osteoblast activity and bone integration [86]. Heat treatment alters the surface characteristics of certain titanium alloys, such as Ti-7Nb-6Al and Ti-13Nb-13Zr, thereby improving cellular compatibility and reducing inflammatory responses [87]. Additionally, the alloy composition and manufacturing process influence biological performance. Ti6Al4V produced through Additive Manufacturing (AM), for instance, demonstrated enhanced metabolic activity in osteoblastic cells, suggesting that processing methods can significantly affect biocompatibility [88]. Ultimately, optimizing both the surface properties and the internal structure of metallic biomaterials is key to ensuring successful performance in biomedical applications.

One of the key challenges in biomedical engineering is the effective regeneration of damaged tissues. To address this, scaffold structures have been developed as crucial tools that support and guide tissue repair processes. Scaffolds are three-dimensional matrices and essential structures that support tissue regeneration, acting as temporary or permanent matrices that promote cellular development and tissue healing. They provide a suitable environment for cell adhesion, proliferation, and differentiation, which are crucial for tissue restoration after damage. Scaffolds are categorized into two main types: permanent scaffolds, which remain stable in the body, and resorbable scaffolds, which are gradually metabolized by the body. They are used to facilitate nutrition transfer and waste excretion, resulting in promoting cellular growth [89] and also serve as a temporary matrix for cellular growth and extracellular matrix formation [90]. Additionally, they help to improve bioactivity and biocompatibility, especially in natural scaffolds, while synthetic scaffolds provide improved mechanical properties.

**Figure 7:**
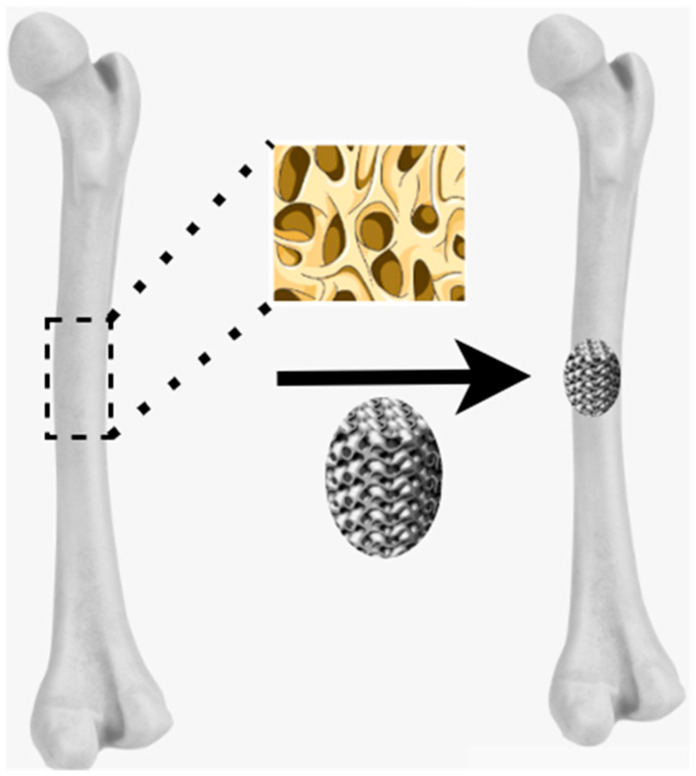
Biomimetic porous titanium scaffold for bone regeneration.

The selection of materials for scaffolds in biomedical applications is crucial, with a particular emphasis on titanium and its porous variations. Porous titanium scaffolds increase bone integration by decreasing stiffness and reducing stress-shielding effects, and also decrease stress shielding and improve bone integration due to their lower stiffness [91]. Although titanium and its porous forms are known for their mechanical benefits, biodegradable materials such as polymers and ceramics are being investigated to develop composite scaffolds that facilitate tissue regeneration while progressively degrading within the body, thereby helping the body heal naturally. Also, they are used to promote osseointegration, allowing effective bonding to nearby bone tissue [92] as shown in Figure 7.

The fabrication of scaffolds usually includes complex processes that can result in significant costs and require a long time. Many fabrication techniques are continuous procedures that use organic solvents, which could negatively impact cell viability and tissue development, which means complicating the production procedure [93]. Although electrospinning techniques are widely used, they need careful control to attain the intended scaffold properties. On the other hand, metal scaffolds used in clinical applications have high stiffness, which can potentially result in stress shielding. This situation arises when the scaffold supports an excess load, reducing the mechanical stimulus needed for bone growth and leading to bone resorption [94]. Additionally, disparity in mechanical properties between the scaffold and the surrounding bone tissue can hinder the healing process and alter the scaffold’s degradation profile [95]. For instance, non-titanium scaffolds, especially synthetic biodegradable polymers, may show poor degradation control. This can cause quick or slower degradation, affecting tissue regeneration and integration. So, to avoid inflammation and insufficient tissue regeneration, the degradation rate must match the rate of new tissue formation [96]. Generally, scaffolds in biomedical engineering play a vital role in tissue regeneration; however, they present several drawbacks, such as intricate fabrication processes, the risk of stress shielding, and insufficient degradation control, particularly in non-titanium scaffolds.

Bone scaffolds in biomedical engineering, especially using AM, offer several advantages that improve tissue engineering and regenerative medicine. They are designed to mimic the natural extracellular matrix, vital for cell attachment and multiplication [97] and serve as a temporary structure for tissue regeneration, providing mechanical support during the healing process. AM techniques allow the production of scaffolds having complex geometry and specific porosity, resulting in improved conditions for the proliferation of cells [98] which is crucial for meeting specific patient anatomical needs and enhancing overall treatment outcomes.

**Figure 8:**
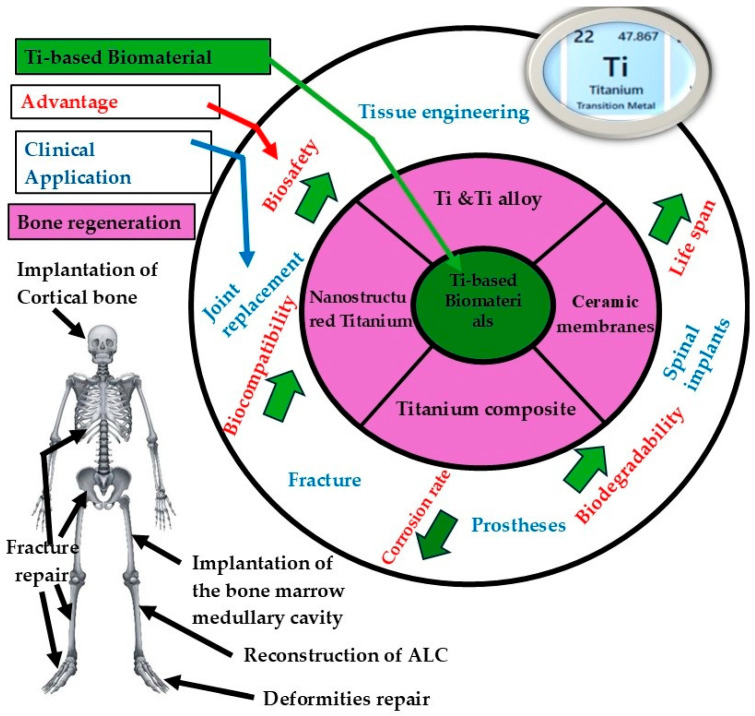
Titanium biomaterials for the human body.

It is clear that the growing elderly population significantly raises the demand for orthopedic devices, specifically in wealthy countries [99,100]. To improve the availability of these devices, significant progress has been made in the field of orthopedic and bone scaffold engineering. In recent years, the number of patients needing to replace failed tissue with artificial implants has increased [101]. These orthopedic implants include fracture fixation, joint replacement (arthroplasty), Spinal implants, and orthopedic prostheses (limb replacement) [102] as shown in Figure 8, the arrows point to the core benefits expected from titanium-based biomaterials: strong biocompatibility, dependable biosafety, reducing corrosion, high mechanical stability, and functional biodegradability. These properties support clinical applications, such as bone regeneration, joint replacement, spinal implants, prostheses, and fracture repair. Generally, this review provides an overview of biomaterials for orthopedic applications, specifically titanium alloys, with a focus on Ti6Al4V. It covers their design, fabrication, modification, and biological evolution to better understand how these materials contribute to bone repair and implant success. In particular, the review intends to:

- Investigate the production and processing techniques used for titanium alloys in orthopedic applications.
- Evaluate 3D-modeled scaffold structures and their production methods.
- Find out how different ways of changing the surface of scaffolds affect their mechanical and biological behavior.
- Summarize findings from in vitro mechanical tests and in vivo assessments of osteogenesis and cellular proliferation.
- Figure out what significant changes have happened and what problems still need to be resolved to make implants work better and improve clinical results.

## 2. Materials and Methods

### 2.1. Material Examined in Reviewed Studies

In this review, titanium and its alloys are recognized as the primary materials of interest, with a focus on titanium-based scaffolds made using 3D printing processes. Titanium has been thoroughly investigated and utilized in the fabrication of bone scaffolds due to its advantageous mechanical strength and excellent biocompatibility. Among them, Ti-6Al-4V stands out as the most widely used alloy. However, alloys such as Ti-35Nb and Ti-35Nb-7Zr-5Ta have been used nowadays due to concerns related to the toxicity of aluminum and vanadium, which have been noted in Ti-6Al-4V [103]. Other alloys, such as Ti-10Nb-10Zr [104], Ti20Nb15Zr [105], and Ti-25Nb-25Zr [106], are also primary titanium alloys used in biomedical applications. Table 3 shows different titanium alloys used in the human body, combined with their main microstructure and references to previous papers. From this Table, it is illustrated that Ti6Al4V is the most versatile alloy used in nearly all implants due to its α + β microstructure, which provides balanced mechanical strength and corrosion resistance. Ti-6Al-7Nb has been usually chosen as a safer alternative due to niobium, which reduces the risk of cytotoxicity associated with vanadium. Although clinical data are limited, β-type alloys such as Ti-13Nb-13Zr and Ti-15Mo, with their reduced elastic modulus and improved biocompatibility, have shown promise for future implant development. Studies have reported that α + β alloys, like Ti6Al4V and Ti6Al7Nb, have a yield strength of 850–950 MPa and elastic moduli of 105–115 GPa, which enables their use in high-load orthopedic applications. On the other hand, β-type alloys (Ti13Nb13Zr, Ti15Mo) have significantly lower moduli, ranging from 65 to 85 GPa, which may limit fatigue resistance but help decrease stress shielding. The overall trend indicates a gradual transition from α + β alloys to β-rich compositions, reflecting the design objective of enhancing biocompatibility through the addition of Nb, Zr, and Mo, while simulating cortical bone strength in the range of 10–30 GPa. This investigation shows different trends in the use of alloys. Due to their higher strength and stability, α + β alloys are preferred for high-load implants; however, β-rich alloys have become the preferred choice for applications requiring less stiffness and lower metal ion release.

The particle size of titanium alloy powders plays a critical role in the production of bone scaffolds using powder-based AM techniques, including Laser Powder Bed Fusion (LPBF), Selective Laser Melting (SLM), and Electron Beam Melting (EBM). Smaller particle sizes result in improved flowability and packing density, which enhances the precision and surface finish of the printed implants. This, in turn, improves the physical and chemical characteristics of titanium alloys, ultimately leading to enhanced scaffold performance in biomedical applications. In a previous paper, various titanium particle sizes were analyzed. Moreover, optimal particle size ensures uniform melting during processes like SLM, resulting in stronger, defect-free structures. However, there is a trade-off: particles that are too fine lead to increased oxidation risk and pose handling challenges. Striking the right balance is key to achieving both high-quality implants and efficient manufacturing.

Although powder particle size is the most critical factor influencing scaffold performance, it remains relevant to the quality of AM processes. Various studies have employed Ti6Al4V powders with size distributions ranging from 15 to 45 μm [141], 20 to 50 μm, and 5 to 50 μm. Pattanayak et al. [142] used Ti6Al4V ELI (Grade 23), which demonstrates superior mechanical strength and biocompatibility due to reduced interstitial elements. While powder characteristics affect flowability and laser interaction, process parameters, such as laser power, scanning speed, and layer thickness, have a greater influence on the resulting microstructure, porosity, and mechanical behavior. However, these parameters are often insufficiently reported in the literature, limiting reproducibility. Search techniques using PubMed and Scopus with targeted keywords (“Additive Manufacturing AND Titanium Alloys AND Bone Scaffolds”; “Mechanical Properties AND Biocompatibility of Titanium Alloy Scaffolds for Bone Regeneration”) corresponding to the focus of the study, and the effectiveness of those studies is summarized in Table 4. Accordingly, only 41% of the reviewed studies reported powder sizes (ranging from 15 to 100 µm), while the remaining 59% omitted this information entirely. Pore architecture, particularly pore size and porosity, plays a critical role in osseointegration and mechanical compatibility. Most of the reviewed studies used pore sizes between 300 and 400 µm [143], which are considered effective for supporting bone ingrowth. However, other works explored a broader range: Deng et al. [144] proposed 650 µm pores with 65% porosity, Wang et al. [145] investigated 800–1000 µm, and Xu et al. [146] used 500 µm with 60% porosity. Chen et al. [147] applied 600 µm pores with 70% porosity, and further analysis by Chen et al. [148] tested multiple pore sizes (500–700 µm) across 60% and 70% porosity levels. Gryko et al. [149] investigated porosity values ranging from 20% to 80% with varying pore geometries. Regarding biological assessment, 72.7% of the studies included both in vivo and in vitro evaluations, 13.6% used in vivo tests only, 9.09% relied on in vitro assays alone, and 4.5% lacked biological evaluation altogether. These findings emphasize the importance of optimizing pore structure and reporting detailed process parameters for developing reliable, biofunctional titanium scaffolds in regenerative medicine.

In Table 4, Ti6Al4V is confirmed as the most frequently used alloy due to its α + β microstructure, which provides a balanced combination of mechanical strength, ductility, and corrosion resistance. AM techniques, particularly SLM and EBM, are frequently used for manufacturing patient-specific scaffolds with defined porosity, which increases osseointegration and bone regeneration. Furthermore, Ti6Al4V’s proven effectiveness across a range of implant types, along with continuous advancements in surface modification and structural optimisation, validate its long-lasting efficacy and adaptability in biomedical applications.

From Table 4, studies reviewed that SLM and EBM were used in the evaluated experiments to manufacture Ti-6Al-4V scaffolds using powder of particle sizes ranging from 5 to 100 µm. A large number of scaffolds had porous architectures, while others used functionally graded designs that consistently outperformed uniform scaffolds in terms of bone ingrowth and mechanical performance. Regarding biological analysis, approximately 73% of the studies included both in vitro and in vivo testing, demonstrating the effectiveness of the scaffold in both physiological and experimental situations.

Based on the analysis of the reported pore designs, pore diameters between 400 and 600 µm were consistently provided. On the other hand, mostly 60–75% porosity of moderate porosity with functional grading improved load distribution and osteointegration, resulting in the most effective results, promoting efficient osteoblast activity, bone regeneration, and integration. In general, scaffolds with smaller holes (less than 400 µm) or larger (more than 600 µm) exhibited less predictable biological reactions or needed functional grading to perform equally. Overall, the data indicate a clear design principle for AM-fabricated Ti-6Al-4V scaffolds: moderate pore sizes, in conjunction with functionally graded structures, yield the most consistent and successful scaffold outcomes for bone healing.

Table 5 summarizes the process parameters reported by the reviewed studies, complementing the information presented in Table 4. The data reveal that, although all studies employed either SLM or EBM techniques to fabricate Ti-6Al-4V scaffolds, the reporting consistency of critical parameters varied greatly. Only 41% of the works clearly specified the powder size, with most falling within the range of 15 to 100 µm, while 59% omitted this key detail. Similarly, several studies failed to disclose essential process inputs such as laser power, scan speed, or layer thickness, which are fundamental to reproducibility and mechanical accuracy of the printed scaffolds. For instance, while studies such as those by Antounian et al. [151], Hindy et al. [152], and Wang et al. [145] reported complete process parameters, others, like Chen et al. [147], provided no data at all.

When compared with Table 4, which primarily emphasizes alloy type, pore geometry, and biological outcomes, Table 5 exposes the gap between design-oriented optimization and process transparency. The most frequently used parameters, including a powder size of 15–45 µm, a laser power of 180–500 W, scan speeds of 300–1250 mm/s, and layer thicknesses of 25–30 µm, represent typical SLM conditions that have proven effective for achieving a uniform microstructure and stable mechanical performance. However, the inconsistency in parameter disclosure limits inter-study comparability and weakens the link between processing conditions, microstructural development, and biological functionality. Integrating the insights from both tables underlines that reliable scaffold performance depends not only on controlled pore architecture but equally on standardized and well-documented fabrication parameters.

### 2.2. Methodology

#### 2.2.1. Method of Collecting Data

This review aims to present current information on different titanium alloys, their use in orthopedic implants, their manufacturing methods, and their performance after implantation. A literature survey was conducted in Scopus and Web of Science to collect research on titanium alloys published between 1 January 2020, and 31 October 2025. In Scopus, the query TITLE-ABS-KEY (“titanium alloy”) was executed, limited to English-language articles in the fields of Materials Science, Engineering, Physics, and Chemistry, yielding 43,254 records. The same search was applied in Web of Science, using (“titanium alloy”) within the Science database, resulting in 25,027 article publications. These results highlight the broad and integrated research perspective on titanium alloy research, encompassing materials science, multidisciplinary, metallurgy, physics, engineering, and chemistry. Relevant studies on titanium alloys for bone implants were selected based on the scaffold properties (size and shape, fabrication method, pore size, and porosity), study features (in vivo and in vitro studies), effectiveness, proliferation, and reported osteogenic outcomes since the objective of this paper is to focus on human bone scaffolds, it concentrates on selected papers that utilize titanium alloy for manufacturing human bone scaffolds. In general, this review employs a research-focused review to evaluate the current advancements in titanium alloys used for bone implants.

#### 2.2.2. Limitation

This work is a narrative review conducted by a single reviewer. Therefore, no formal assessment of study quality or bias was performed. The review emphasizes Ti-6Al-4V because it is the most extensively studied alloy; however, other titanium systems received less attention due to the scarcity of comprehensive research on their performance. The summary and discussion of results focused on describing and comparing reported findings rather than performing quantitative analysis. Finally, the literature search covered studies from 2020 to 2025.

#### 2.2.3. Method of Bone Scaffold Preparation

In tissue engineering, designing bone scaffolds involves the use of computational simulations and modeling tools to refine structural parameters and results of that, ensuring the scaffolds achieve the mechanical strength and biocompatibility essential for successful bone regeneration [165]. The use of computer-aided design (CAD) and simulation technologies plays a crucial role in the development of bone scaffolds by enabling the creation of detailed three-dimensional models, which can subsequently be analyzed through FEM to assess mechanical performance and structural reliability [166,167]. These tools facilitate the integration of mechanical, chemical, and biological parameters into scaffold design, supporting the improvement of pore geometry and overall architecture for enhanced functionality in a biological environment [168].

The improvement of scaffold architecture and material composition in the design of titanium alloy implants is guided by several theoretical concepts. To prevent stress shielding, which can lead to bone loss, the mechanical approach aims to minimize the stiffness mismatch between the implant and natural bone. This mismatch is commonly quantified using the stress shielding factor (SSF), which is the ratio of the stress carried by the bone with the scaffold to that of native bone alone.

$$\mathrm{SSF}=\frac{\sigma^{\mathrm{with}\;\mathrm{scaffold}}}{\sigma^{\mathrm{natural}}}=\frac{E}{E+E^{\mathrm{eff}}}$$

where E is the elastic modulus of the bone, and E^eff^ is the effective elastic modulus of the scaffold. Effective scaffold stiffness is approximated as

E^eff^ = ETi(1 − P)^n^

where P is the porosity fraction and n is the empirical exponent (typically 1.5–2 for open cell structures). Recent studies show that increasing porosity in Ti6Al4V scaffolds reduces the elastic modulus [149], thereby bringing the mechanical properties closer to those of natural bone. The relationship between porosity and elastic modulus has been studied, highlighting their importance in minimizing stress shielding [169,170]. This is generally achieved by modifying the lattice design, porosity, and pore geometry to accomplish an effective elastic modulus that closely matches that of cortical bone (E ≈ E^eff^), ensuring physiological load transfer [171]. Under an applied uniaxial load F, the stress distribution between the scaffold and the bone is then governed by the equation,

$$\sigma_{b}=\frac{E}{E+E^{\mathrm{eff}}}\sigma \;\mathrm{applied},\;\sigma_{s}=\frac{E}{E+E^{\mathrm{eff}}}\sigma \;\mathrm{applied}$$

From a biological perspective, parameters such as surface roughness, surface energy, and pore interconnectivity, which influence protein adsorption, cell adhesion, and nutrient exchange, are crucial for effective osseointegration. Theoretical approaches that connect surface roughness (R_a_), surface energy (γ), and adhesion strength (Wadh) elucidate how energetic and topographical components interact to regulate cell material interactions. The Young Dupré equation states that the adhesion work between a liquid (or biological cell medium) and a solid surface is as follows:

Wadh = Y_LV_ (1 + cos θ)

where Y_LV_ is the liquid vapor surface energy and θ is the contact angle. This means that a smaller ϑ indicates stronger adhesion. On the other hand, according to the Wenzel relation, surface roughness enhances the apparent surface energy.

Cos θ = rcos θ

where r > 1 is the roughness factor. As roughness increases, the effective contact area expands, resulting in increased protein adsorption and enhanced cell adherence [172]. To improve the attachment of osteoblasts and ensure that osseointegration remains stable over time, it is essential to optimize surface roughness and energy. In addition, alloying elements such as zirconium, tantalum, and niobium are included to stabilize the β-phase and improve corrosion resistance and biocompatibility. Depending on these theoretical analyses, titanium alloy scaffolds for bone regeneration have been designed and selected.

**Figure 9:**
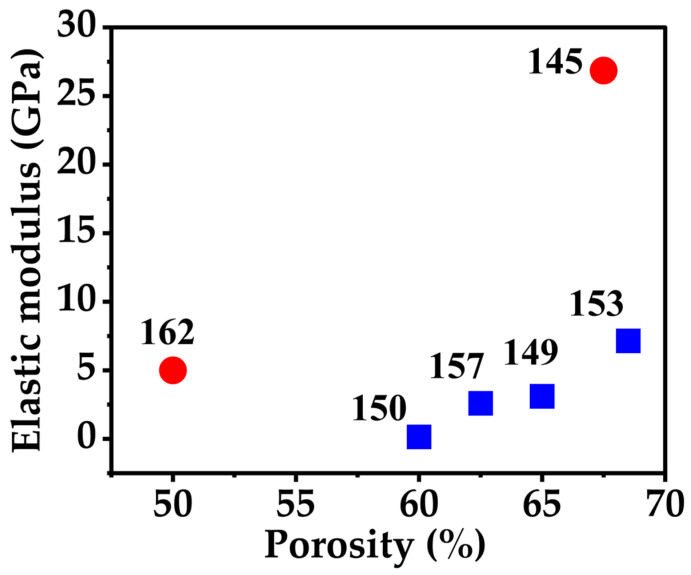
Ashby plot illustrating the relationship between porosity and elastic modulus for various titanium scaffold architectures investigated by researchers.

The relation between porosity and elastic modulus is crucial in determining how titanium scaffolds behave mechanically. Similar to the usual pattern in cellular metals, where a higher void content lowers load-bearing capacity, stiffness decreases as the internal pore volume increases. This inverse correlation is shown in Figure 9 for several scaffold architectures. Both diamond and BCC lattices [145,153] transmit stress well through interconnected struts, and they show a higher elastic modulus at moderate porosities. Circular and hollow prism geometries [144,146] are less stiff, which is similar to the characteristics of cancellous bone and reduces stress shielding. In the middle range are gyroid designs [149] and triply periodic minimal surfaces [158], which combine excellent pore interconnectivity with sufficient strength. Together, these findings demonstrate how the mechanical response of titanium scaffolds can be fine-tuned to more closely resemble the behavior of native bone by varying their internal geometry and porosity.

Individual reference sets fitting correlations change due to variations in scaffold design, manufacturing routes, and testing conditions. In Figure 9, the red circle points representing references [145,162] correspond to scaffolds with denser architectures and stronger stress-transmission paths, resulting in a higher elastic modulus trend. The blue rectangles, taken from references [149,150,153,157] represent more open, less dense structures with a comparatively lower elastic modulus. These structural differences naturally produce distinct slopes on the Ashby plot, illustrating how scaffold design critically influences mechanical behavior at comparable porosity levels.

**Figure 10:**
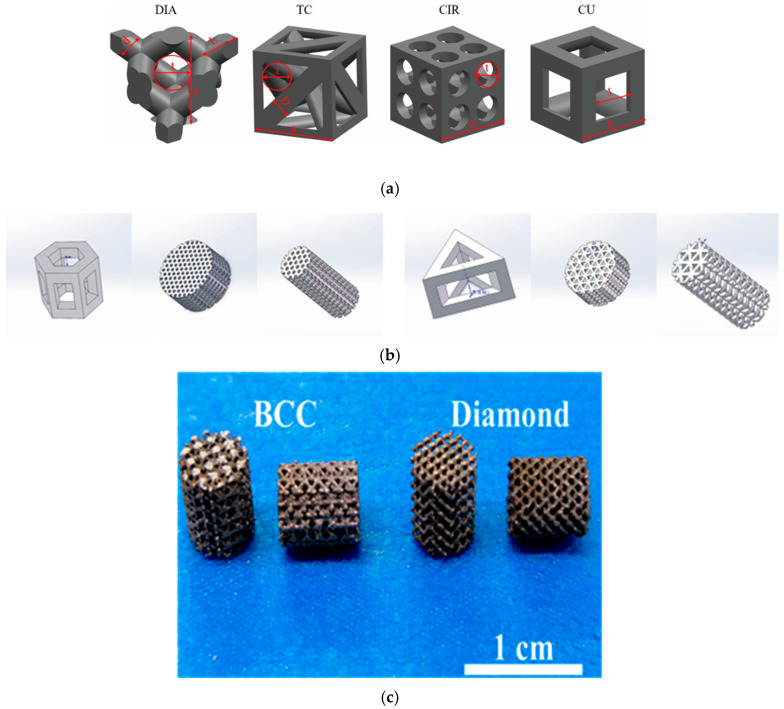
The unit cell scaffold had been used (**a**) Porous scaffolds with different unit cell designs: DIA (Diamond), TC (Truncated Cube), CIR (Circular), and CU (Cube) [144], (**b**) a hollow hexagonal prism and a hollow triangular prism [146]. (**c**) BCC and diamond scaffold [153].

Several studies have demonstrated that scaffold geometry significantly influences biological and mechanical outcomes. Deng et al. [144] evaluated four common porous architectures: Diamond, Truncated cube, Circular, and Cube (DIA, TC, CIR, CU) as illustrated in Figure 10a and observed that diamond-shaped pore structures, fabricated from Ti6Al4V, notably promoted bone ingrowth and cellular proliferation in both in vivo and quantitative models. Gryko et al. [149] investigated the effects of various internal geometries, spherical, octagonal prism, cube, and triangular prism, and concluded, based on finite element simulations, that scaffolds with 40% porosity and optimized geometries exhibit mechanical characteristics comparable to natural bone. Xu et al. [146] assessed cylindrical scaffolds with hollow hexagonal and triangular prism shapes, reporting that the hexagonal structure enhanced osteogenic activity and bone formation more effectively, as illustrated in Figure 10b. Similarly, Chen et al. [147] conducted comprehensive in vitro and in vivo evaluations on disc-, bullet-, and cylindrical-shaped scaffolds and found that porous Ti6Al4V cages with varying AM angles improved cell proliferation and bone tissue integration. Wang et al. [145] designed and fabricated scaffolds featuring TPMS architectures, which were subsequently analyzed to evaluate their structural and mechanical properties. Accordingly, seven types of diamond shapes, R8, R9, R10, P8, P9, P10, and NP, were prepared for investigation, and found that Large-pore Ti6Al4V scaffolds with irregular pore architectures improve vascularized bone regeneration, and P10 design shows the optimal balance between mechanical strength and biological performance. While Sun et al. [153] use BCC and diamond shapes based on different strut sizes, as illustrated in Figure 10c, and found that bone ingrowth in additive-manufactured titanium scaffolds, particularly in cortical bone-filled pores, enhances mechanical properties and long-term structural performance. However, Li et al. [158] prepared typical Primitive, Diamond, and Gyroid (P, D, and G surfaces) scaffolds resulting in TPMS models on Ti6Al4V scaffolds promoting early osteointegration and bone ingrowth, which is used to improve bone tissue engineering by reducing stress shielding and improving osteogenesis. Furthermore, Hindy et al. [141] generate gradient lattice structures with a dense core and a surrounding porous lattice. While Chen et al. [148] designed complex shapes and porous structures, they found that Ti6Al4V ELI scaffolds with 500 μm pore size and 60% porosity increase cell proliferation, osteogenesis, and bone growth in both in vitro and in vivo tests.

Extensive research has highlighted the critical role of scaffold architecture in facilitating effective bone regeneration. Titanium alloy scaffolds manufactured through additive techniques, featuring carefully engineered pore morphologies such as diamond-shaped configurations, hexagonal arrangements, and TPMS, have been shown to markedly promote osteointegration, vascularization, and new bone formation. These specific structural designs achieve a favorable balance between mechanical durability and biological performance, thereby improving both the mechanical support and the cellular environment necessary for successful bone tissue engineering. By tailoring pore geometry and dimensions, these scaffolds enhance nutrient diffusion and cell proliferation without compromising the structural integrity required for load-bearing applications [144,173].

#### 2.2.4. Method of Manufacturing Bone Scaffold

Several manufacturing techniques have been developed to enable the use of titanium and its alloys in bone implantation. The process of manufacturing titanium alloys encompasses several crucial phases that transform raw materials into high-performance materials suitable for various applications, particularly in the aerospace, medical, and industrial sectors. While the production of titanium alloys must be adapted to suit particular biomedical applications and desired material characteristics, typical fabrication techniques include forging, casting, powder metallurgy, and AM. However, due to its advantages, like reducing material costs and improving efficiency by producing components with low waste [174] creation of complex geometries [175] customizes microstructures and mechanical properties by precisely controlling processing settings, improving performance [173] over traditional manufacturing methods, AM is preferable for titanium alloy production. Complex geometries and customized microstructures are required to enhance the performance of titanium components in various applications.

SLM and EBM are powder bed fusion techniques used in the AM of titanium scaffolds. SLM operates with a laser in an inert gas environment, providing high precision, while EBM uses an electron beam under vacuum, offering faster processing and lower residual stress [176]. Both enable the fabrication of porous structures suitable for medical implants. Those AM methods use distinct energy sources, laser and electron beam, respectively, and have become essential technologies for fabricating biomedical scaffolds. SLM, in particular, has shown notable advantages in achieving high structural resolution, surface finish, and favorable mechanical characteristics. Its capacity to fabricate complex porous geometries supports load-bearing functionality and facilitates osteointegration. Furthermore, the integration of rapid prototyping allows for iterative optimization during scaffold development, which is crucial in dynamic clinical environments [177].

Through SLM, it is possible to produce anatomically customized implants tailored to individual patients, thereby improving surgical precision and clinical outcomes [178]. This method is suitable for generating dense structures that meet the mechanical demands of orthopedic load-bearing applications [179]. Both LPBF, which includes SLM and EBM, enable the fabrication of scaffolds with biomimetic architectures. These architectures closely replicate the structure of natural bone, promoting better tissue integration and enhancing biological performance in orthopedic applications [171]. Additionally, these technologies utilize medical imaging data like Computed Tomography or Magnetic Resonance Imaging (CT/MRI) to design patient-specific implants, improving overall functionality and biocompatibility [180]. Innovations such as hybrid volumetric energy density control in LPBF have further optimized scaffold performance by achieving a balance between mechanical integrity and controlled porosity [181]. Despite these advancements, challenges such as heterogeneity in mechanical properties and porosity distribution remain. Nevertheless, LPBF techniques, including SLML, remain highly effective for fabricating titanium-based scaffolds. Their advantages include high geometric precision, design flexibility, efficient material use, and strong potential for clinical integration.

**Figure 11:**
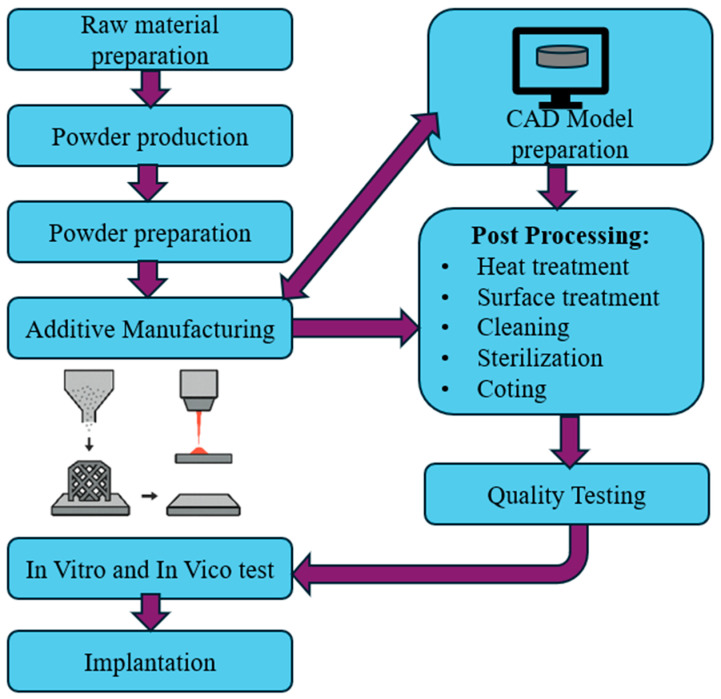
Process of manufacturing of titanium alloy.

Accordingly, the majority of the tests evaluated were conducted using SLM, and the detailed manufacturing process is illustrated in Figure 11. Li et al. [158] point out that SLM has been thoroughly applied for the production of Ti6Al4V scaffolds. Additionally, researchers have used SLM for the fabrication of Ti6Al4V discs, cages, and porous structures [146,147,150,158]. Furthermore, various alternative manufacturing methods have been used to create scaffolds with preferred characteristics, for example, Wang et al. [145] used EBM to create porous Ti6Al4V scaffolds, while Sun et al. [153] used LPBF technology.

## 3. Discussion of Results

### 3.1. Implantation

This review provides an evaluation and summary of current research on the design and production of bone scaffolds utilizing titanium alloys, as well as their impact on the biocompatibility and bifunctionality of proposed systems. The design stage of scaffolds is crucial for bone regeneration and functionality, influencing aspects such as cell infiltration, osteogenic differentiation, and overall healing outcomes. Studies show that improving scaffold design, such as channels’ dimensions, their configuration, and surface characteristics, can significantly improve the regeneration of bone tissue. Entezari et al. [182] suggest that circular measuring diameter of approximately 0.9 mm improves bone formation more effectively than smaller (0.3 mm) or larger (1.5 mm). Also, hierarchical porous scaffolds featuring nanofibrous structures promote cell proliferation and direct osteogenic differentiation, closely resembling the natural extracellular matrix [183]. Additionally, scaffolds that adjust to various bone defects enhance integration with adjacent tissues, thereby improving repair outcomes. In real cases, an in vivo test of implants was used to evaluate biocompatibility, osseointegration, and mechanical stability in a living biological environment.

**Figure 12:**
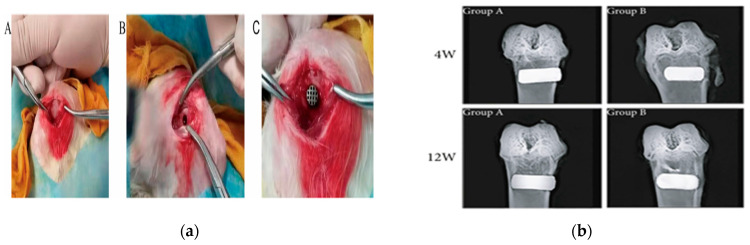

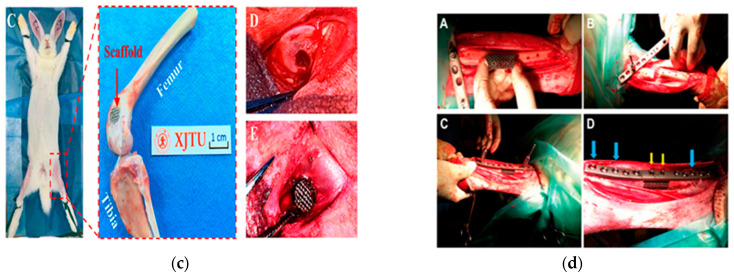
Implantation in different animals (**a**) Implantation in the white rabbit; (A) Exposure of the distal lateral condyle of the rabbit femur. (B) Creation of a cylindrical bone defect of 5 mm × 8 mm in the lateral condyle. (C) Implantation of the 3D-printed Ti6Al4V scaffold into the bone defect [144]. (**b**) Results of X-ray evaluation of femur samples post-scaffold implantation; Group A and B at 4 W and 12 W (in a rabbit femur defect model) show different healing rates: Group B displays faster bone ingrowth and stronger pore filling, while Group A shows slower, more gradual regeneration [146]. (**c**) the scaffold and location of the implanted scaffolds (C) Surgical site showing the rabbit femur for scaffold implantation. (D) A bone defect was created in the lateral femoral condyle. (E) A titanium scaffold was placed into the defect for bone ingrowth [153]. (**d**) Photographs showing the surgical implantation of a titanium scaffold (experimental group) for the tibia; (A) Placement of the 3D-printed porous Ti6Al4V scaffold into the critical tibial defect. (B) Alignment of the titanium locking plate to stabilise the reconstructed segment. (C) Fixation of the plate and scaffold with screws to secure the defect reconstruction. (D) Final construct showing all screws in place (blue arrows) and full scaffold integration into the stabilised tibia (yellow arrows). [157].

Achieving reliable osseointegration remains a critical factor for the long-term success of titanium-based implants, particularly in the early stages following implantation [184]. To assess the biological performance and integration potential of such implants, various animal models have been employed in preclinical research. Small animals like New Zealand white rabbits [144,146,153,155] indicated in Figure 12a–c and Sprague-Dawley (SD) rats [148] are commonly used due to their cost-effectiveness and ease of handling, offering valuable insights into initial bone response, tissue compatibility, and early-stage bone formation. Larger animal models, such as Beagle dogs [147], provide more clinically relevant data on mechanical loading and long-term implant stability. At the same time, sheeps tibia are used for large bone defects [157] for evaluating scaffold performance in critical-sized bone defects due to their anatomical and physiological similarities to human bone, as shown in Figure 12d.

Across these studies, titanium scaffolds consistently show excellent osseointegration, tissue compatibility, and structural stability [144,146,148,153,157]. Additionally, optimal pore geometry and customized porosity improve bone tissue regeneration [182,183]. Due to differences in animal models, implantation sites, and healing durations, evidence on which specific scaffold shapes and pore shapes increase effectiveness varies. Additionally, most investigations evaluate only short-term integration, which limits insight into long-term mechanical and biological stability under physiological loads [147,157]. Overall, while architectural design promotes regeneration, evidence quality is weak due to model and follow-up differences.

Rather than material inconsistencies, differences in bone metabolism, loading conditions, and physiological behavior are the primary causes of variation in implant durability throughout animal models. The mechanical forces [185] and joint stresses that occur in larger animals, such as sheep or dogs, can accelerate wear or fatigue at the implant bone interface. Small animals, such as rats and rabbits, on the other hand, experience less mechanical stimulation but higher rates of bone turnover and healing, which can often result in an overestimation of early osseointegration performance [186]. Bone mineral density and remodeling response are also influenced by diet and activity levels; active or calcium-rich species typically promote more extended implant durability and greater fixation. Therefore, rather than the intrinsic performance of the titanium material itself, differences in durability between models result from a combination of biomechanical demand, metabolic rate, and lifestyle-related factors.

### 3.2. In Vivo Evaluation of Scaffold Effectiveness

#### 3.2.1. Effectiveness Evaluation

Evaluating the efficacy of titanium implants is essential for analyzing their performance regarding biocompatibility, osseointegration, mechanical stability, and long-term clinical results. Accordingly, Deng et al. [144] evaluated the bone regeneration capacity of cylindrical porous titanium scaffolds implanted in rabbit femurs. These scaffolds featured a uniform pore size of 650 µm and a porosity of 65%, parameters chosen to enhance bone ingrowth. Their findings revealed a significantly increased Bone Tissue to Total Volume (BT/TV) ratio compared to non-porous titanium implants, reflecting improved osseointegration, as similarly noted by Chen et al. [148] observing reduced bone ingrowth in scaffolds with smaller pore sizes and lower porosity levels, underscoring the critical role of scaffold architecture, especially pore size and interconnectivity, in facilitating effective bone tissue integration.

While Chen et al. [147] evaluated the volume of newly produced bone within the pore space of the SLM Ti6Al4V implant and found that increment from 11.89% at 4 weeks to 15.85% at 12 weeks of implantation, and finally, concluded as there is higher bone ingrowth compared to other implants that have relatively lower porosity (50–60%), showing that, porous Ti6Al4V cages clinically applicability. Additionally, Liu et al. [161] evaluated the volume fraction of newly formed bone within porous scaffolds, observing continuous bone ingrowth over time. Their simulation results illustrated that as bone tissue progressively fills the scaffold pores, the pore size decreases. This finding highlights the dynamic interaction between scaffold architecture and bone regeneration, suggesting that while initial pore geometry supports tissue infiltration, the reduction in pore size during healing may affect nutrient diffusion and mechanical properties, emphasizing the need for optimized pore design to maintain long-term scaffold functionality.

On the other hand, Xu et al. [146] found that Micro-Computed Tomography (Micro-CT) results showed increased bone mass in hexagonal and triangular scaffolds at week 12 compared to week 4. While fluorescence imaging showed reduced material contact signals, suggesting that hexagonal scaffolds improve bone formation. Also, the result of optical microscopy showed no significant difference in new bone production between groups at week 4 (*p* > 0.05). However, hexagonal scaffolds had considerably higher new bone area than triangular ones by week 12 (*p* < 0.05). While Wang et al. [145] suggest that as the hole size increases, bone tissue growth into the scaffold eventually increases in area and volume. This is true for porous and non-porous scaffolds, leading to a progressive rise in angiogenesis. Additionally, Li et al. [158] observed bone development in both porous and nonporous groups five weeks post-implantation in pigs (micro-CT) and found that active osteogenesis was observed surrounding all porous scaffolds; however, a significant gap was observed around the nonporous scaffolds. This indicates a better stability of the bone-implant contact in TPMS-based porous scaffolds.

Hindy et al. [152] investigated the biocompatibility of titanium-based 3D-printed functionally graded porous scaffold materials using Periodontal Ligament Stem Cells (PDLSCs). Flow cytometric analysis revealed that PDLSCs expressed mesenchymal stem cell surface markers CD90, CD44, and CD105, while lacking expression of hematopoietic markers CD34 and CD45. These results confirm the mesenchymal phenotype of the cells and demonstrate their compatibility with the tested scaffolds, underscoring the potential of these materials for supporting stem cell-mediated tissue regeneration.

**Figure 13:**
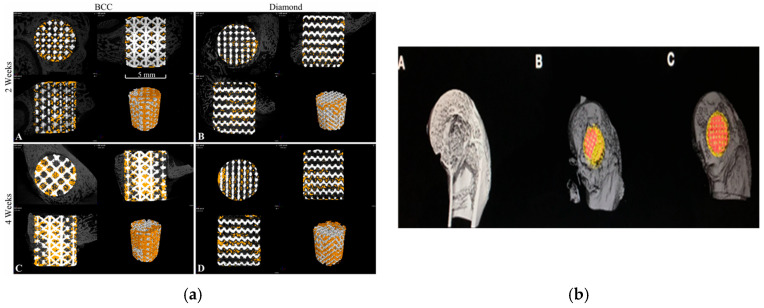
Micro-CT analysis (**a**) Micro-CT images and reconstructed 3D models of the retrieved scaffolds with representative volume elements (RVEs) of bone-cement composite (BCC) and diamond at two and four weeks post-operatively (white: scaffolds; orange: bone tissue); (A) BCC scaffold after 2 weeks shows early bone ingrowth beginning to occupy internal pores. (B) Diamond scaffold after 2 weeks shows limited early bone deposition mainly along the inner walls. (C) BCC scaffold after 4 weeks shows extensive bone filling and strong internal integration. (D) Diamond scaffold after 4 weeks shows increased bone formation but less filling than the BCC design [153]. (**b**) Images of 3D reconstruction using Micro-CT on the femoral defect; (A) CT scan showing the femoral condyle defect before implantation. (B) Post-implant scan displaying the 3D-printed titanium scaffold positioned in the defect. (C) Follow-up scan showing bone growth surrounding and integrating with the implanted scaffold [155].

Sun et al. [153] made the comparison between BCC and diamond shape scaffolds, and the results show that the bone volume fractions in the BCC scaffolds are significantly greater than those of the diamond scaffolds (Figure 13a). While Zhong et al. [155] suggest a greater formation of new bone within the Volume Of Interest (VOI) around the Polydopamine-Coated 3D-printed porous titanium (PDA-3D PPT) implants compared to other groups, and the Bone-Implant contact (BIC) of PDA-3D PPT was higher (*p* < 0.05) compared to the 3D-printed porous titanium (3D PPT) group (Figure 13b) based on micro-CT 3D reconstruction results. Additionally, Chen et al. [148] suggests that Ti6Al4V ELI scaffolds with a 500 μm pore size and 60% porosity exhibit optimum bone ingrowth for osteointegration; on the other hand, Liu et al. [161] analyzed the comparative investigation of the bone-in-growth process in the high-stress-stimulus group, assessing discrepancies between experimental findings and simulation results, and a μ-CT study found that mechanical loading significantly improved the growth of bones in 3D-printed Ti-alloy scaffolds. Finally, for large defect bone, Crovace et al. [157] prepared an investigation on sheep, and after three months post-removal of the titanium support plates, the sheep were euthanised, and the tibiae were taken out for histological evaluation, which shows that large animal critical bone defects ensure quick loading of the animal and facilitate better anatomical regrowth of the bone defect.

In vivo studies consistently demonstrate that porous titanium scaffolds with pore sizes between 500 and 650 μm and a porosity of 60 to 65% promote enhanced bone ingrowth and osseointegration [144,148,153,161]. However, there are disagreements on the most effective lattice design (BCC, hexagonal, TPMS), which is context-dependent and affected by implantation conditions [146,153,155]. Although the current evidence is convincing, it is primarily based on short-term studies involving small animal models, which creates uncertainty regarding their effectiveness for larger human-scale defects.

#### 3.2.2. Osteogenic and Cell Proliferation

The osteogenic properties of titanium alloy implants are vital for facilitating bone growth and integration, making them a crucial element in their effectiveness in orthopedic applications. Xu et al. [146] suggest that two groups of scaffolds (a hollow hexagonal prism and a hollow triangular prism) showed nontoxicity to the cells and had excellent biocompatibility. Additionally, quantitative analysis of alkaline phosphatase activity in cells on scaffolds indicates that the osteogenic capacity of the scaffold in the hexagonal group was higher than that in the triangular group. While Wang et al. [187] investigated the biological performance of nano-copper-bearing 316L stainless steel and found that it promotes osteogenic differentiation without impairing cell proliferation. The incorporation of nano-sized copper particles into the stainless steel matrix accelerated callus formation and enhanced fracture healing, indicating its potential for orthopedic applications requiring both mechanical strength and osteoinductive properties.

On the other hand, Marrow-Derived Mesenchymal Stem Cell (BMSC) adhesion and proliferation assays are essential in evaluating the biocompatibility and osteogenic capacity of biomaterials, offering insights into their efficacy in facilitating bone tissue regeneration. Hindy et al. [152] assessed the proliferation of human periodontal ligament stem cells (hPDLSCs) on SLM fabricated Ti-6Al-4V constructs using the MTT assay at 24 and 72 h post-seeding. The results demonstrated significantly higher cell proliferation on the dense titanium samples compared to porous ones at both time points (*p* < 0.05), highlighting the influence of surface density on cellular behavior and suggesting favorable biocompatibility of SLM-processed Ti-6Al-4V for dental and orthopedic applications. Also, cell-to-cell and cell-to-surface connections were visible. While the results of live/dead staining indicate strong cell adhesion to the constructs, the interconnected cells have a standard form, and a few apoptotic cells. While Wang et al. [145] found from live and dead cell analysis, larger pores increased cell connectivity and proliferation, and irregular porous scaffolds demonstrated higher cell growth compared to their regular counterparts with identical pore sizes. At the same time, SEM images showed that BMSCs adhered to the scaffold and exhibited a spindle-like morphology, with an increase in cell attachment corresponding to larger pore sizes.

On the other hand, Chen et al. [147] investigated 3D-printed porous Ti6Al4V cages designed with optimized architectural features, such as increased internal pore angles, and demonstrated that these geometries significantly enhanced cell proliferation and osteogenic activity. In vivo evaluation using a Beagle tibia model revealed that the engineered scaffolds supported bone ingrowth comparable to that of conventional commercial implants. While Tan et al. [188] indicated that TiCu/Ti-Cu-N coated scaffolds improved cell adhesion and proliferation relative to Ti6Al4V, showing good biocompatibility and facilitating osteoporosis fracture healing. Additionally, Gou et al. [143] suggest that TiCu/Ti-Cu-N coated 3D-printed Ti6Al4V scaffolds significantly enhanced hBMSC adhesion, proliferation, and recruitment, thereby allowing effective bone regeneration in vivo. While according to Crovace et al. [157] 3D biomimetic porous Ti6Al4V ELI scaffolds significantly facilitated bone regeneration and osseointegration in large tibial defects in sheep, promoting rapid functional recovery and stable implant integration. Generally, Surface morphology, alloy composition, and pore geometry all have significant effects on cell proliferation and osteogenic differentiation [143,145,147,187,188]. However, findings differ concerning the most suitable pore size or coating to balance biological and mechanical performance [145,147]. Some studies choose denser surfaces for early proliferation, while others prefer porous, coated forms for long-term osteogenesis. Despite strong evidence of improved osteogenic potential, there are relatively few comparative and standardized in vivo studies.

### 3.3. In Vitro Evaluation of Scaffold Effectiveness

#### Mechanical Test Result

The mechanical testing of bone scaffolds is necessary to evaluate their effectiveness in vitro, since it evaluates their capacity to mimic the mechanical properties of native bone and facilitate cellular activities vital for tissue engineering. Accordingly, Morgan et al. [189] found that site-specific modulus density correlations affect in vitro bone mechanical integrity assessments by compressing trabecular bone at various anatomical sites, and found that the elastic modulus of bone trabeculae varies from 0.1 to 4.5 GPa, and the yield strength of the proximal tibia and proximal femur ranges from 0.56 to 55.3MPa. While Deng et al. [144] used Ti6Al4V of 15 to 45 µm particle size, found that the yield strengths of the three structures out of four examined, DIA, CIR, CU, and Tetrahedral close-packed (TC), exceed this range, and the elastic modulus of all four structures ranges from 1.9 to 4.2, showing promising results for implant. On the Other hand, Wang et al. [145] used regular and irregular pores and concluded that the elastic modulus of the regular pore structure scaffold declined as the pore size increased, with the minimum value recorded at R10 (10.37 ± 0.25 GPa). In comparison, the elastic modulus of the irregular pore structure scaffold decreased as the pore size increased, with the lowest value recorded at P10 (15.53 ± 0.55 GPa). Additionally, the compressive strength of the regular pore structure scaffolds was from 50 MPa to 150 MPa, while the compressive strength of the regular pore structure scaffolds ranged between 100 MPa and 200 MPa. While Sun et al. [153] found that the elastic modulus and yield strength were 3.23 GPa and 91.50 MPa, respectively. From those papers, it is shown that titanium scaffolds can mimic human cancellous bone [144,145,153,189]. Nevertheless, viewpoints differ on the ideal porosity level that balances biological performance and stiffness. It is hard to compare results since the studies vary in fabrication methods, pore design, and particle characteristics. Moreover, most experiments examine only static compression, without accounting for fatigue or cyclic loading, leaving the long-term mechanical reliability uncertain.

### 3.4. Result of Surface Modification on Bone Scaffold

In biomedical engineering, modifying the surface characteristics of materials has become a fundamental approach to overcoming limitations related to biological compatibility and functionality. Although many materials possess suitable mechanical properties, their interaction with living tissues may be suboptimal. Surface modification addresses these issues by refining surface attributes, such as texture, chemical composition, and surface energy, while preserving the core structural integrity. This process enhances cellular behavior, promotes tissue integration, reduces the potential for microbial contamination, and fine-tunes degradation rates. Consequently, surface engineering plays a key role in advancing the clinical performance and reliability of medical implants and scaffolding systems used in regenerative therapies [190]. It is essential to note that the type of surface treatment applied to titanium-based scaffolds has a substantial impact on their biocompatibility. On the other hand, surface topography, chemistry, and wettability affect osteoblast attachment and differentiation, as well as protein adsorption and cell-material interactions [191]. Optimized surface treatments enhance osseointegration by creating micro- and nanoscale characteristics that promote bone ingrowth, thereby reducing the risk of unfavorable tissue reactions.

To enhance the biological performance of titanium scaffolds, numerous surface modification strategies have been developed. These techniques are generally grouped into mechanical, physical, chemical, and biological categories. Physical modifications such as grit blasting and acid etching introduce surface roughness at the microscale, which facilitates improved cell adhesion. Chemical alterations, including anodic oxidation and micro-arc oxidation, adjust both the surface composition and morphology to boost bone-bonding capabilities. Meanwhile, biological modifications often apply bioactive coatings, like hydroxyapatite or magnesium-based layers, to emulate the natural bone matrix and stimulate osteogenic cell responses and bone tissue regeneration [192,193]. Additionally, surface treatments must critically address the fundamental physical properties that are crucial to the long-term functionality of bone implants, thereby promoting bioactivity. The combined effects of wear resistance, surface wettability, and coating adhesion heavily influence how well an implant interacts with the surrounding bone tissue.

**Figure 14:**
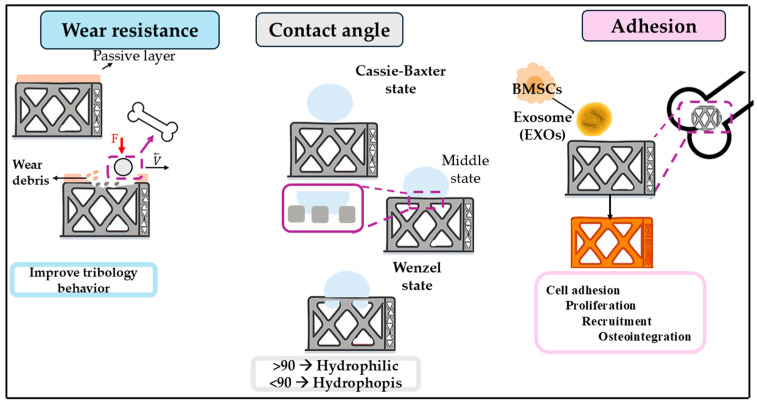
Interactive Role of Surface Wettability, Coating Adhesion, and Wear Resistance in Bone Integration (F represents the frictional force acting on the surface, and V indicates the sliding velocity during wear).

The contact angle is especially vital, as it affects surface wettability, wear resistance, and bonding strength. Paxton et al. [194] reported that porous titanium scaffolds show several types of wetting behaviors, including Wenzel and Cassie-Baxter states, and that proper contact-angle measurement is essential for correctly determining hydrophilicity and its influence on fluid and cell interaction. Wear resistance is critical for minimizing particle release since inflammation at the bone-implant interface is a major contributor to implant failure. Optimizing the surface’s tribological effectiveness, for instance, through the addition of a stable oxide layer or reinforced coating, can efficiently reduce debris formation and improve the lifespan of implants. A suitable contact angle is crucial for osseointegration, as it facilitates cell attachment and protein adsorption. Strong adhesion, on the other hand, ensures that coatings remain intact under mechanical loading conditions, minimizing delamination and improving bone integration, cell recruitment, and proliferation (Figure 14). The mechanical stability and biological performance of titanium scaffolds could decline if their exterior characteristics are not appropriately modified [195,196]. Studies have shown that altering the physical surface properties of titanium implants promotes the growth and proliferation of bone marrow. However, under certain conditions, especially when employing dual surface alteration techniques, mechanical characteristics such as compressive strength may be substantially reduced. To maintain both mechanical reliability and biological activity, a successful surface design requires striking a balance between hydrophilicity, wear resistance, and coating adhesion.

**Figure 15:**
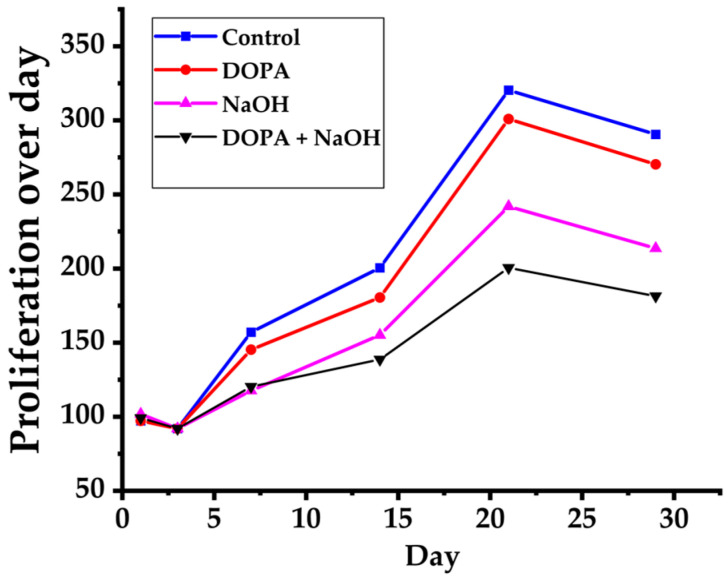
Proliferation curves of osteoclasts propagated on the porous titanium scaffold (Reproduced from Ma et al., Biomimetics 2024, 9, 423, under the terms of the Creative Commons Attribution (CC BY) license [197]).

Accordingly, Ma et al. [197] demonstrated that the coatings (NaOH, DOPA, and a combination of NaOH and Dopamine (DOPA)) on Ti6Al4V alloy and their impacts on the scaffold’s mechanical properties, as shown in Table 6, surface treatment of those porous scaffolds results in small changes to compressive strength (150 ± 6.7 MPa for NaOH, 155.8 ± 7.1 MPa for DOPA and 141.8 ± 4.6 MPa for NaOH + DOPA), but results in substantial improvements to osteoblas viability and growth. These results demonstrate that, while the mechanical behavior is largely equivalent among the treatments, the added benefit primarily derives from biological factors. Although the combination of NaOH and DOPA treatment results in a slight reduction in compressive strength (141.8 ± 4.6 MPa compared to 149.7 ± 4.9 MPa for the control), coating analysis indicates that Ti-DOPA and Ti-NaOH coatings exhibit a slight increase in mechanical performance compared to the uncoated control. All coatings significantly improve osteoblast adhesion and proliferation despite these small, subtle changes in mechanical variations, with dual treatment providing the most significant biological response. These results suggest that coating selection has a greater impact on biological outcomes than on mechanical behavior, and single coatings (NaOH or DOPA) are effective. In contrast, a dual NaOH + DOPA approach might fail slightly from a mechanical standpoint and requires further optimization. For proliferation analysis after 21 days of in vitro testing, the surfaces of the porous scaffolds in all groups were completely covered with cells, resulting in effective cell attachment and proliferation on the scaffolds, as shown in Figure 15.

Similarly, Mofazali et al. [198] reported that the Alginate–Tragacanth (S) scaffold (AT (S)) exhibited the highest compressive strength, measured at 40.7 ± 0.02 MPa. But upon modification with 5% gelatin and 5% alginate (5G5A) scaffold (5Gel-5Alg (S)), the compressive strength decreased to 38.01 ± 0.09 MPa, and further reduced to 36.02 ± 0.87 MPa when modified with 8% gelatin and 2% alginate scaffolds (8Gel-2Alg (S)). Despite this reduction in mechanical strength, SEM results revealed enhanced cell spreading on the modified surface, indicating improved surface bioactivity and suitability for bone tissue engineering.

**Figure 16:**
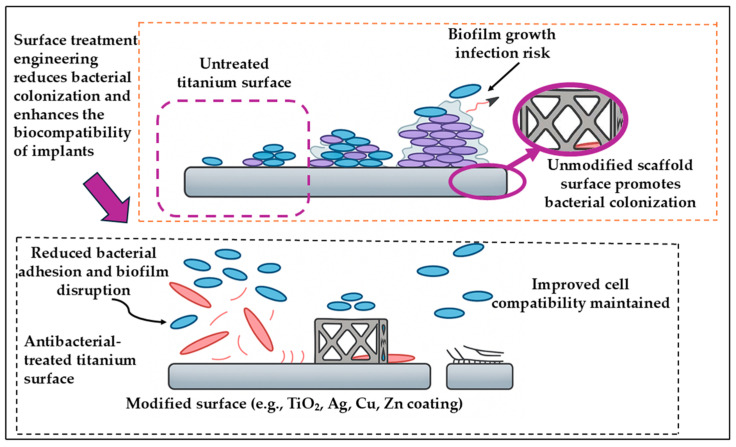
Schematic illustration of antibacterial surface modification mechanisms on titanium scaffolds; **Colour description**: The blue oval figures represent individual bacteria that are still free-moving before firmly attaching to the metal surface. The purple layered clusters depict groups of microbes that have already organised into a biofilm on untreated titanium. The red, rod-like shapes indicate bacteria that have been damaged or eliminated by the antibacterial coating. Grey elements indicate the titanium scaffold itself, both before and after surface treatment. The pink or purple-outlined area highlights sections of an unmodified scaffold where bacterial buildup is likely to occur. The light reddish shading on the lower titanium surface indicates the presence of an added antimicrobial layer (such as TiO_2_, silver, copper, or zinc). The blue cell-like shapes above the treated surface represent healthy host cells, emphasizing that the modified coating continues to support good biological compatibility. (Adapted from Gallo et al., 2014, with permission under the CC BY license [199]).

Although the primary goal of surface modification is to enhance bone bonding, it also plays a crucial role in protecting implants from bacterial infection, a problem that scaffold research often overlooks. As Gallo et al. [199], titanium alloys can easily attract bacterial colonies if their surface chemistry or microscopic texture is not properly adjusted. Infection remains one of the primary reasons implants fail, so refining the surface is not just about biocompatibility, but also about controlling infection. Several methods have been investigated, including the development of nanotube oxide films, the formation of photocatalytic TiO_2_ layers, and the addition of small amounts of antibacterial metals like zinc, copper, or silver. These methods support effective cell attachment while minimizing bacterial development. While active coatings combat bacteria directly by releasing antimicrobial chemicals, passive modifications often reduce bacterial adhesion by altering roughness or wettability. Combining both effects with bioactive design enhances the surface’s tissue-growth-supportive properties and resistance to infection, thereby improving implant integration and long-term reliability. Figure 16 illustrates a simplified representation of antibacterial surface modification methods applied to titanium scaffolds. By enabling bacterial adhesion and biofilm formation, the untreated surface (top) reduces implant stability and increases the risk of infection. After surface modification, antibacterial coatings such as TiO_2_, Ag, Cu, or Zn decrease bacterial adhesion and biofilm formation while maintaining cell compatibility and promoting osseointegration.

The current study has demonstrated that surface treatments can impact the antibacterial properties of titanium implants, various modification methods, such as photocatalytic TiO_2_ surfaces, nanotubular oxide layers, and coatings with silver, zinc, or copper ions, can prevent bacterial growth while maintaining the antibacterial surface design of scaffold engineering bacterial growth while preserving good cytocompatibility. Biofilm development and bacterial adherence are significant factors contributing to implant failure [200]. Implant acceptance and long-term success can be enhanced by the antibacterial surface design of scaffold engineering.

Chemical surface modification techniques are designed to change the surface properties of materials, improving their functionality and performance for specific applications. According to Wilk et al. [201], anodic oxidation under optimized voltage and hydrodynamic conditions significantly increased the corrosion resistance of the Ti6Al4V alloy, determined by reduced corrosion current densities in Ringer’s solution. Additionally, Poquillon et al. [202] showed that thermal oxidation at the temperature range of 450 to 600 °C led to the creation of an oxygen-enriched zone in the Ti-6Al-4V alloy. This modification notably impacted its creep behaviour and decreased ductility, regardless of the affected zone, indicating a reduction of merely 5% of the cross-section. While Casadebaigt et al. [203] suggested that the high-temperature oxidation of the Ti-6Al-4V alloy produced through AM led to the development of a brittle layer enriched with oxygen. This phenomenon had a significant impact on the mechanical properties, particularly the tensile strength, due to oxygen diffusion into the alloy.

On the other hand, Gu et al. [204] showed that incorporation of BMP-2 into a biomimetic CaP coating on 3D-printed titanium scaffolds significantly improved mandibular bicortical bone formation in a beagle dog model, confirmed by an increase in bone volume fraction and bone-to-implant contact. In contrast, Zhang et al. [205] found that titanium scaffolds coated with a bio-surface, designed to mimic cancellous bone structures, significantly improved osteogenic differentiation and bone formation, while also exhibiting strong antibacterial properties in both in vitro and in vivo models. These results confirm the dual purpose of surface modification, which is essential for achieving long-term implant success by controlling bacterial adherence and promoting osseointegration. Similarly, surface modifications, such as plasma treatment, crosslinking with agents, and functional group grafting, significantly enhance scaffold properties, resulting in improved cell attachment, proliferation, and bone regeneration in tissue engineering applications. While Woźniak et al. [206] found that the combination of laser texturing and electrospun PCL/TiO_2_ nanofiber coatings on Ti-6Al-4V ELI titanium significantly improved surface wettability, corrosion resistance, and biocompatibility, thereby increasing its suitability for biomedical applications. In general, researchers report that surface modifications enhance cell adhesion, osteoblast activity, and antimicrobial behavior. However, mechanical strength or ductility may be negatively affected by dual or high-temperature treatments [197,201,203]. There is limited information on the long-term mechanical performance and durability, and most investigations focus on biological outcomes [198]. Overall, there is strong evidence for biological enhancement; nevertheless, a more in-depth study of its impacts on longevity and biomechanics is required.

### 3.5. Commercial Titanium Scaffold System

Commercial titanium scaffold systems demonstrate how research on Am is being applied in medical applications. Devices approved by the FDA, such as patient-specific EBM implants and DePuy titanium mesh cages, have already demonstrated mechanical stability and safety [207,208]. However, challenging regulatory pathways, limited long-term follow-up data, and unclear cost–benefit profiles continue to hinder translation at scale [207,209]. There are incompatible associations with each manufacturing method. For instance, powder metallurgy enables scalable, cost-effective production [17,210], while EBM offers greater powder recyclability and reduced contamination [211], and SLM enables high geometric precision but requires substantial post-processing [212,213]. Even though they improve osseointegration, surface modification methods, such as growth factor delivery systems and bioactive glass coatings, pose additional manufacturing and regulatory challenges [154,214,215]. The osteogenic potential of new alloy compositions, such as Ti-Ta-Nb-Zr, is more effective than that of traditional Ti6Al4V [216]. Direct comparison with standard treatments is limited by ongoing information gaps, especially the absence of multicenter studies, long-term registries, and health-economic evaluations [207,209,217]. Coordinated translational frameworks that include standardized preclinical standards, modular scaffold design libraries that balance manufacturability and customization, and early inclusion of sustainability evaluations will be essential to progress. The use of predictive modeling and AI-driven design is an example of new methods that can speed up patient-specific optimization [208,213].

Several commercial systems have demonstrated the clinical use of porous titanium technologies. The trabecular-like, biomimetic lattice used by EIT Cellular Titanium promotes bone ingrowth and speeds up fusion. Two FDA-cleared porous titanium implants, the Tritanium PL Cage (FDA 510 (k) K152304) and the InTice-C Porous Ti Cervical Interbody System (FDA 510 (k) K173832), have demonstrated reliable mechanical strength and strong osteoconductive properties in spinal fusion procedures. Other commercially available systems, including Modulus, HEDRON, Ghost, IB3D, TrellOss, and Zyston, further highlight the adaptability of additive manufacturing for producing interconnected porous structures that enhance load distribution and bone ingrowth. Osseointegrative scaffold systems can enhance future translation by facilitating iterative validation, providing real-time clinical feedback, and enabling rapid optimisation. Reassemblable scaffold systems can improve future tr, osseointegration by facilitating iterative validation, real-time clinical feedback, and quick optimisation of patient-specific implant design [218].

### 3.6. Challenges and Future Directions in Metal Alloy Bone Scaffold Development

Two main issues in titanium-based scaffold design are still being addressed by current research: insufficient early osseointegration and the mismatch between implant stiffness and native bone. Even though titanium and its alloys are strong and resistant to corrosion, their elastic modulus (110–120 GPa) remains much greater than that of cortical bone (10–30 GPa), which causes stress shielding and slows down bone resorption. It has been shown that increasing porosity via AM decreases effective stiffness and improves bone ingrowth [219]. Research indicates that while porosity levels of 60–70% and pore sizes of 300–700 µm optimize biological response [220]. excessive porosity decreases fatigue strength and reduces long-term load-bearing capacity. As a result, maintaining structural balance through controlled pore geometry, interconnectivity, and graded stiffness remains a critical design problem.

The composition of the materials heavily influences this balance. Ti-6Al-4V, the most commonly used α+β alloy, continues to dominate scaffold fabrication due to its favourable strength and corrosion profile [103,144,151,154,155,158,159,160,161,162,163,164,165,166,167]. Concerns about cytotoxicity associated with vanadium and aluminium have drawn attention to β-rich alloys, including Ti-35Nb, Ti-35Nb-7Zr-5Ta, Ti-10Nb-10Zr, and Ti-25Nb-25Zr [103,215,221,222]. Although β-phase titanium alloys help match bone elasticity and generally support good cellular response, this often comes with a drop in tensile and fatigue strength. Table 7 consolidates numerical results from the reviewed papers, illustrating how different alloying elements influence β-phase stability and corrosion behavior. It provides a straightforward comparison of how composition influences both the mechanical performance and chemical durability of alloys used in scaffold design. Strong β-stabilisers, such as Mo, Nb, and Zr, promote the material toward a stable or fully β structure and shift the corrosion potential to more positive values, indicating higher resistance to electrochemical attack, as shown in Table 7. Poorer corrosion resistance is indicated by alloys with weaker β-stabilizing components, which typically maintain a higher α phase and exhibit more negative corrosion potentials.

On the other hand, the mechanical-biological trade-off cannot be sufficiently addressed by alloy design alone, which is also affected by particle dimension variations (15–50 µm) [144,145,147] These factors, in turn, impact mechanical reliability and biocompatibility, which are influenced by differences in surface polish and melt uniformity. Therefore, optimizing powder and processing conditions is crucial, as smaller particles increase oxidation risk but enhance precision.

Complementary benefits have been documented by surface modification. Early cell adhesion and antibacterial activity are substantially improved by processes such as anodisation, sol–gel coating, and atomic layer deposition [192,193,194,195,196,200]. But the durability of these coatings and the potential harmful effects of antibacterial drugs remain unclear. The next improvement could come from a mixed-methods approach that combines improved β-alloy scaffolds with bioactive surface modifications [232].

Future studies should focus on integrated, multiscale optimization rather than isolated testing. Pore design can be guided by computational modeling and FEA [149], and such structures can be precisely converted into patient-specific implants using AM. FEA lacks biological processes such as osteogenesis and resorption, instead recording the mechanical response of scaffolds. To more precisely predict scaffold performance during bone healing, future models should incorporate mechanics with tissue growth and remodel kinetics, according to studies by Lekszycki and dell’Isola [233] and Allena et al. [234].

When such a mechanical design is combined with biologically active coatings and real-time degradation monitoring, scaffolds that respond to the healing process can be produced. To identify reliable structure-property-function relationships and expedite the clinical translation of titanium alloy scaffolds, it will be crucial to establish standardized experimental frameworks that link mechanical, corrosion, and biological data under identical conditions. To strengthen these acceptable international standards that promote clinical translation and reproducibility, they have been incorporated into only a few of the evaluated studies. The need to align alloy processing and testing with these frameworks is demonstrated by studies such as Nikiel et al. [74], followed ASTM F136 [235], while both Yu et al. [164] and Wang et al. [60] used ASTM F2924 [236]. Das and Balla [175] incorporated ASTM F3302 [237], ISO, and ASTM 52900 guidelines in their work. Increased use of ISO 5832 [238] and ASTM standards such as F136, F2924, and F3302 ensures reliable mechanical performance, consistent material quality, and a more transparent path from research to clinical and industrial applications.

Despite the development of ISO 10,993 and ISO 52900 [239] frameworks, their uniform implementation across titanium implant research is still limited, as recent analyses [240,241] emphasize the necessity of standards-driven approaches for ensuring the consistency, biocompatibility validation, and traceability of AM systems, ultimately improving the transition of laboratory findings into approved clinical implant solutions.

## 4. Conclusions

The presented studies collectively demonstrate that titanium alloys, such as Ti6Al4V and TiNbZr, or implant subsidiary materials like Ti-Ta, provide better results than commercially pure titanium in terms of mechanical stability, corrosion resistance, and biocompatibility. Nb, Zr, or Ta additives in the compositions result in a decrease in the elastic modulus, as well as improved bone compatibility, and suppress the stress shielding effect. AM and isostatic pressing enable more precise control of porosity and microstructure, enhancing fatigue strength and promoting tissue ingrowth compared to conventional casting. Regarding surface modification methods, atomic layer deposition, sol–gel coatings, and anodisation are particularly effective at promoting osteogenic adhesion while reducing bacterial development. Combinedly, these results highlight that the most balanced mechanical and physiological outcomes are achieved by combining optimized alloy composition, precise structural design, and bioactive surface engineering.

When considered collectively, these results highlight the need for more comprehensive study approaches that integrate alloy composition, processing conditions, and biological outcomes. Rather than analyzing each factor separately, future studies should aim to measure how material design and surface structure together affect mechanical behavior and cell performance. Additionally, although the review identified key gaps and future challenges, many studies are limited to short-term animal models, lack standardized reporting of process parameters, and fail to assess long-term mechanical and biological stability under physiological loads sufficiently. Future research should therefore emphasize multi-scale modeling, standardized experimental validation, and clinical translation of titanium scaffold technologies. Ultimately, this type of comparative, multidisciplinary approach is crucial for advancing titanium-based implants toward more consistent and durable clinical outcomes.

## Figures and Tables

**Table 2:** Mechanical properties of titanium alloy used for biomedical applications [66,70,71,72,73,74,75,76,77,78,79,80,81].

S/No.	Grade	Name	Structure Type	Young’s Modulus (GPa)	Tensile Strength (MPa)	Fatigue Strength (MPa)	Hardness (HV)	Elongation (%)
CP-Ti								
1	Grade 1	Titanium CP1	alpha (α)	103	240	160	120	24–28
2	Grade 2	Titanium CP2	alpha (α)	103	345	240	160	20
3	Garde 3	Titanium CP3	alpha (α)	103	450	310	200	18
4	Grade 4	Titanium CP4	alpha (α)	103	550	340	250	15
aluminium vanadium titanium alloy								
5	Grade 5	Ti-6Al-4V	α + β-type	113–120	895–965	510–560	330–370	10–14
6	Grade 8	Ti-7.35Al-1Mo-1V	Near α	110–120	690–830	550–620	300–350	10–15
7	Grade 9	Ti-3Al-2.5V	α + β-type	110–115	620–900	400–480	300–360	10–15
8	Grade 18	Ti-3Al-2.5V-0.05Pd	α + β-type	107–110	620–740	450–500	300–350	15–17
9	Grade 19	Ti-8V-6Cr-4Mo-4Zr-3Al	β-type	103	793–1000	500–650	330–390	8–12
10	Grade 20	Ti-8V-6Cr-4Zr-4Mo-3Al-0.06Pd		105–115	795–100		340–400	8
11	Grade 23	Ti-6Al-4 V ELI	α + β-type	101–110	860–980	510–620	320–260	10
12	Grade 24	Ti-6Al-4V-0.06Pd	α + β-type	110–115	860–980			10–14
13	Grade 25	Ti-6Al-4V-0.5Ni-0.06Pd	α + β-type	105–115	860–980			10–15
14	Grade 28	Ti-3Al-2.5V-0.5Ru	near α-type	105–107	620–630		290–340	10–18
15	Grade 29	Ti-6AL-4V-0.1Ru ELI	α + β-type	110	890		320–370	16
Others, titanium alloy								
16	Grade 6	Ti-5Al-2.5Sn	α-type	110–120	828–972	290–350	290	10–16
17	Grade 7	Ti-0.15Pd	α-type	110	485		250–330	12–20
18	Grade 10	Ti-11.5Mo-6Zr-4.5Sn	β-type	80–100	730–850	380–400	330–380	13
19	Grade 11	Ti-0.15Pd	α-type	103	345		250–330	20–37
20	Grade 12	Ti-0.3Mo-0.8Ni	near α-type	103	483–607		280–350	18–22
21	Grade 13	Ti-0.5Ni-0.05Ru	α-type	110–120	275			20–24
22	Grade 14	Ti-0.5Ni-0.05Ru	α-type	105	410			12–20
22	Grade 15	Ti-0.5Ni-0.05Ru	α + β type	110	484			19
23	Grade 16	Ti-0.06Pd	α-type	115–110	345–483		160–230	20–30
24	Grade 17	Ti-0.06Pd	α-type	105–115	241–345		160–230	24–37
25	Grade 21	Ti-15Mo-3Nb-3Al-0.2Si	β-type	90–100	793		280–350	15
26	Grade 26	Ti-0.1Ru	α-type	105–115	345			20
27	Grade 27	Ti-0.1Ru	α-type	105–115	300			20–30
28	Grade 36	55Ti-45Nb	β-type	45–60	500–700			22
Non-grade biomedical Ti-alloys								
29		Ti-6Al-7Nb	α + β-type	110–114	580–710		300–350	8.1–15
30		Ti-13Nb-13Zr	β-type	60–81	900–1005	490–550		12–13
31		Ti-35Nb-7Zr-5Ta	β-type	40–55	597	260–300		19–20
32		Ti-29Nb-13Ta-4.6Zr	β-type	60–80	912			13
33		Ti-24Nb-4Zr-7.9Sn	β-type	45–55	830			12–15
34		Ti-15Mo	β-type	78	874			21
36		Ti-15Mo-2.8Nb-0.2Si	β-type	83	990			16–18
37		Ti-16Nb-10Hf	β-type	81	852			11
38		Ti–25Pd–5Cr	β-type	110–120	880			5

**Table 3:** Application of titanium alloys in humans and their structural composition.

Titanium Alloy	Microstructure	Category of Use	Uses	Source
Pure Titanium,	α	Orthopedic implant	Parts of the joint (stems, cups, etc.), meshes, artificial bones, and fixation instruments	[56,104,105,106,107,108,109,110,111,112]
Ti6Al4V	α + β			
Ti6Al7Nb	α + β			
Ti13Nb13Zr	β			
Ti15Mo	β			
Pure titanium	α	Cardiovascular devices	Medical supplies such as ventricular assist devices, implantable defibrillators, clips, guidewires, catheters, and heart valves.	[113,114,115,116,117,118,119,120,121,122]
Ti15Mo	β			
Ti6Al4V	α + β			
Ti6Al7N	α + β			
Pure titanium	α	Spinal implants	Fixing devices, discs, and cages	[123,124,125,126,127,128,129,130]
Ti6Al4V	α + β			
Pure titanium	α	Trauma devices	Fixing plates, screws, and rods	[69,131,132,133,134]
Ti6Al7Nb	α + β			
Ti6Al4V	α + β			
Pure titanium	α	Soft tissue implant	Fixation apparatus, hernia devices, breast reconstruction prosthesis	[135,136,137,138,139,140]
Ti6Al7Nb	α + β			
Ti6Al4V	α + β			

**Table 4:** List of Ti6Al4V scaffolds and their effectiveness.

Author, Date	Particle Size	Ti-Alloy Used	Vivo/Vitro Test	S-Manufacturing	Effectiveness After Implantation/of the Article	Positive Innovation
Tilton et al., 2021 [150]	_	Ti-6Al-4V (Grade 5) spherical powder	Vitro	EBM	The patient-specific AM prosthesis showed enough biomechanical strength to avoid.	Patient-specific AM. prostheses could effectively restore substantial bone defects following tumor excision.
Antounian et al., 2024 [151]	15–45 μm	Gas-atomized Ti-6Al-4V Grade 23	Vitro and vivo	SLM	The implant was successfully integrated with the bone, and after 14 months, an X-ray was taken.	The patient’s functional status showed improvement, and limb shortening was efficiently minimized.
Hindy et al., 2020 [152]	5–50 μm	spherical Ti-6Al-4V powder.	Vitro	SLM	Demonstrate the feasibility of using 3D printed functionally	Functionally graded samples with dense cores had a good match of Young’s
Deng et al., 2021 [144]	15–45 µm	Ti6-Al-4V	Vivo	SLM	After the removal of the scaffold from the implant, it was found that a greater.	Uses 3D printing technology to produce functionally graded porous titanium alloys, which increases.
Wang et al., 2021 [145]	20–50 µm	porous Ti-6Al-4V	vitro and vivo	EBM	Ti6Al4V scaffolds for vascularized bone regeneration were tested for biocompatibility and bone ingrowth	generating better vascularization and osseointegration for better bone regeneration in orthopedic applications
Gryko et al., 2022 [149]	_	Ti-6Al-4V porosity and pore geometry	Focuses on FEA	_	FEA simulates the mechanical properties of different pores	Improve scaffold design by showing how computational modeling may optimize
Xu et al., 2022 [146]	_	Ti-6Al-4V powder	Vivo and Vitro	SLM	In vitro and in vivo investigations have shown that 3D-printed Ti6Al4V scaffolds enhance osteoblast activity, bone regeneration, and osseointegration.	Using VEFG/BMP 2 microspheres for sequential growth factor release with a 3D-printed porous titanium alloy scaffold to improve bone.
Sun et al., 2022 [153]	_	Ti-6Al-4V powder	Vivo	LPBF	In vivo tests and FEM show that additive-manufactured Ti6Al4V	show that cortical bone ingrowth causes significant mechanical changes in additive-manufactured Ti6Al4V scaffolds, offering a predictive basis for optimizing porous implant designs.
Zhang et al., 2021 [154]	36 µm	Ti-6Al-4V powder	Vivo and Vitro	SLM	Bioactive glass and mesoporous bioactive glass-coated 3D-printed Ti-6Al-4V structures facilitate bone regeneration by promoting cell growth, bone conduction, and angiogenesis.	Mesoporous bioactive glass coatings on 3D-printed Ti-6Al-4V scaffolds enhance bone regeneration by promoting both osteogenesis and angiogenesis through careful structural design.
Chen et al., 2019 [147]	30 μm	Ti-6Al-4V alloy powder	Vivo and Vitro	SLM	uses 3D-printed Ti6Al4V porous cages to show that surface modifications increase cellular behavior in vitro and bone ingrowth in vivo.	Adjusting additive angles promotes biocompatibility, osseointegration, and bone structure in 3D-printed porous Ti6Al4V scaffolds.
Zhong et.al., 2020 [155]	_	Ti-6Al-4V	Vivo and vitro	SLM	Polydopamine-coated 3D-printed Ti-6Al-4V implants enhance cell adhesion, osteogenesis, bone regeneration, and osteointegration in vitro and in vivo.	A polydopamine coating on 3D-printed Ti-6Al-4V implants improves biocompatibility and osteointegration.
Guo et al., 2020 [143]	_	Ti-6Al-4V	Vivo and Vitro	SLM	The success of TiCu/Ti-Cu-N-coated 3D-printed Ti6Al4V scaffolds in recruiting BMSCs for bone regeneration.	TiCu/Ti-Cu-N-coated 3D-printed Ti6Al4V scaffolds were developed to recruit BMSCs and promote osteogenic differentiation for bone regeneration.
Ma et al., 2021 [156]	_	Ti-6Al-4V alloy and gelatin	Vivo and Vitro	SLM	In orthopaedic applications, this hybrid scaffold may improve bone defect repair and tissue regeneration.	Biomimetic hybrid scaffold combining Ti-6Al-4V’s mechanical strength with GelMA’s biocompatibility and bioactivity.
Crovace et al., 2020 [157]	45–70 µm	Ti-6Al-4V	Vivo and vitro	EBM	Over one year, sheep models via EBM-sintered Ti6Al4V scaffolds showed better healing, defect restoration, and mechanical stability.	The scaffolds are intended to match bone mechanical properties, increasing biomechanical stability and healing.
Li et al., 2019 [158]	_	Ti-6Al-4V	Vivo and Vitro	SLM	Enhanced scaffold porosity and mechanics improve osseointegration and early bone healing over traditional designs.	TPMS-designed Ti6Al4V scaffolds enhance porosity and mechanics, promoting osteointegration and bone regeneration.
Fan et al., 2020 [159]	_	Ti-6Al-4V	Vivo and Vitro	SLM	Successful bone regeneration and osseointegration were found in animal models.	Electroactive BaTiO_3_-coated Ti6Al4V scaffolds with LIPUS stimulation enhance osteogenesis and osseointegration.
Ragone et al., 2020 [160]	_	Ti-6Al-4V (Grade 5)	Vivo and Vitro	SLM	In animal models, the scaffold enhances bone ingrowth and integration, thereby supporting effective repair of bone defects.	AM-fabricated randomized trabecular titanium scaffolds enhance osseointegration and bone healing.
Chen et al., 2020 [148]	22–51 μm	Ti-6Al-4V ELI, (Grade 23)	Vivo and vitro	SLM	Improved osteogenesis and bone ingrowth in Ti6Al4V ELI scaffolds with customized pore sizes and porosity.	Optimized pore size and porosity of Ti6Al4V ELI scaffolds via SLM improve scaffold performance.
Liu et al., 2020 [161]	15–45 μm	Ti-6Al-4V	Vivo and vitro	SLM	3D-printed Ti-6Al-4V scaffolds enable bone ingrowth and osseointegration, with mechanical stimulation boosting bone formation.	3D-printed Ti-6Al-4V scaffolds with tailored mechanics enhance bone ingrowth and osseointegration.
Li et al., 2019 [162]	_	Ti-6Al-4V	Vivo and vitro	SLM	PDA-coated 3D-printed Ti-6Al-4V scaffolds improve osteogenesis, cell adhesion, and osseointegration in vitro and in vivo	Polydopamine (PDA) coating on 3D-printed Ti-6Al-4V scaffolds boosts osteogenesis, cell adhesion, and bone regeneration.
Luan et al., 2019 [163]	_	Ti-6Al-4V	Vivo and vitro	EBM	Pore size and porosity in Ti-6Al-4V scaffolds enhance cell proliferation, osteogenesis, bone regeneration, and osseointegration.	Optimizing the pore size and porosity of Ti-6Al-4V scaffolds enhances bone regeneration and osteogenesis.
Yu et al., 2023 [164]	_	Ti-6Al-4V	vitro	SLM	Titanium scaffold with various kinds of shapes for improved cell growth and mechanical strength	Developed functionally graded titanium scaffolds with tailored porosity for mechanical strength and biological integration.

**Table 5:** Reporting of Key Additive Manufacturing Parameters (Powder Size, Laser Power, Scan Speed, and Layer Thickness) in Reviewed Ti-6Al-4V Scaffold Studies.

Study	Powder Size	Laser Power	Scan Speed	Layer Thickness	Powder Size Reported (Y/N)	Laser Power Reported (Y/N)	Scan Speed Reported (Y/N)	Layer Thickness Reported (Y/N)
Antounian et al. [151]	15–45 µm	80 W	900 mm/s	25 µm	Y	Y	Y	Y
Hindy et al. [152]	5–50 µm	175 W	2000 mm/s	30 µm	Y	Y	Y	Y
Wang et al. [145]	45–100 μm	720 W	800, 900, and 1000 μm,	30 µm	Y	Y	Y	Y
Deng et al. [144]	15–45 µm	500 W	300 mm/s	30 µm	Y	Y	Y	Y
Zhang et al. [154]	36 μm	400 W	300 mm/s	30 µm	Y	Y	Y	Y
Chen et al. [147]	30 μm	-	-	-	Y	N	N	N
Crovac et al. [157]	45–70 µm	-	-	70 µm	Y	N	N	Y
Chen et al. [148]	22–51 μm	240 W	240 mm/s	30 µm	Y	Y	Y	Y
Liu et al. [161]	15–45 µm	180 W	1250 mm/s	-	Y	Y	Y	N

Note(s): Y/N—Yes or No.

**Table 6:** Compressive strength of scaffolds as a result of coating application (Reproduced from Ma et al., Biomimetics 2024, 9, 423, under the terms of the Creative Commons Attribution (CC BY) license) [197].

Specimens	Compressive Strength (MPa)
Control	149.7 ± 4.9
Ti-NaOH	150.5 ± 6.7
Ti-DOPA	155.8 ± 7.1
Ti-NaOH + DOPA	141.8 ± 4.6

**Table 7:** Elemental Composition and Its Influence on β-Phase Stability and Corrosion Resistance in Titanium Alloys.

Alloy System	Major Alloying Element Composition wt (%)	B Phase Stabilizing Strength (wt)	Beta Phase Stability	Corrosion Potential (V vs. SCE)	Reference
Ti10Mo	Mo: 10.0	10	Metastable β + α″	−0.15 to −0.10	Moshokoa et al. [223]
Ti-15Mo-5In	Mo: 15.0, In: 5.0	16.6	Predominantly β + α + α″	−0.10 to −0.06	Romero-Resendiz et al. [224]
Ti-35Nb-6Mo	Nb: 35.0, Mo: 6.0	15.7	β phase (increases with processing)	−0.08 to −0.03	Gouvêa et al. [225]
Ti-20Zr-15Mo	Zr: 20.0, Mo: 15.0	16.8	Single β (after 800 °C anneal)	−0.06 to −0.02	Yue et al. [226]
Ti-6Mo-5V-3Al-2Fe	Mo: 6.0, V: 5.0, Al: 3.0, Fe: 2.0	11.8	Metastable β + secondary α	−0.10 to −0.05	Zhang et al. [227]
Ti-6Mo-5V-3Al-2Fe-2Zr	Mo: 6.0, V: 5.0, Al: 3.0, Fe: 2.0, Zr: 2.0	12.8	β + variable α	−0.08 to −0.04	Zhang et al. [228]
Ti55Al40Mo5	Al: 40.0, Mo: 5.0	6	bcc(Ti) single phase	−0.05 to 0.00	Zeng et al. [229]
Ti-0.3Mo-0.8Ni (TA10)	Mo: 0.3, Ni: 0.8	1	α phase dominant	−0.35 to −0.30	Wang et al. [230]
Ti-Cr-Mn	Cr: 5–8, Mn: 2–4	8–12	Near/metastable β + α″ + ω	−0.20 to −0.12	Hong et al. [231]

wt—weight. V vs. SCE—the corrosion potential measured in volts relative to the saturated calomel electrode.

## Data Availability

No new data were created or analyzed in this study. Data sharing is not applicable to this article.

## Abbreviations

AD	Anno Domini
AM	Additive Manufacturing
ASTM	American Society for Testing and Materials
BCC	Body-Centered-Cubic
BIC	Bone-Implant contact
BMP-2	Bone Morphogenetic Protein-2
BMSC	Marrow–Derived Mesenchymal Stem Cells
BT/TV	Bone Tissue to Total Volume ratio
C	Circular
CAD	Computer-Aided Design
CD34	Cluster of Differentiation 34
CD44	Cluster of Differentiation 44
CD45	Cluster of Differentiation 45
CD90	Cluster of Differentiation 90 (Thy-1)
CD105	Cluster of Differentiation 105 (Endoglin)
CP-Ti	Commercially Pure Titanium
CT/MRI	Computed Tomography or Magnetic Resonance Imaging
CU	Cube
DIA	Diamond
DOPA	Dopamine
ELI	Extra Low Interstitials, Grade 23
FEA	Finite Element Analysis
G	Gyroid
HCP	Hexagonal-close-packed
HPDLSCs	Human Periodontal Ligament Stem Cells
ISO	International Organization for Standardizationfacturing
LPBF	Laser Powder Bed Fusion
Micro-CT	Micro-Computed Tomography
MTT assay	(3-(4,5-dimethylthiazol-2-yl)-2,5-diphenyltetrazolium bromide)
P	Primitive
PDA-3D PPT	Polydopamine-coated 3D-printed porous titanium
PDLSCs	Periodontal Ligament Stem Cells
PE	Polyethylene
PLA	Polylactic acid
SLM	Selective Laser Melting
TPMS	Triply Periodic Minimal Surface
TC	Truncated cube
TC	Tetrahedral close-packed (TC)
VEGF	Vascular Endothelial Growth Factor
3D	Three-Dimensional
3D PPT	3D-printed porous titanium
5Gel-5Alg (S)	5% gelatin and 5% alginate (5G5A) scaffold
8Gel-2Alg (S)	8% gelatin and 2% alginate scaffolds
